# Next-generation sequencing of immunoglobulin gene rearrangements for clonality assessment: a technical feasibility study by EuroClonality-NGS

**DOI:** 10.1038/s41375-019-0508-7

**Published:** 2019-06-13

**Authors:** Blanca Scheijen, Ruud W. J. Meijers, Jos Rijntjes, Michèle Y. van der Klift, Markus Möbs, Julia Steinhilber, Tomas Reigl, Michiel van den Brand, Michaela Kotrová, Julia-Marie Ritter, Mark A. Catherwood, Kostas Stamatopoulos, Monika Brüggemann, Frédéric Davi, Nikos Darzentas, Christiane Pott, Falko Fend, Michael Hummel, Anton W. Langerak, Patricia J. T. A. Groenen, on behalf of the EuroClonality-NGS Working Group

**Affiliations:** 1 Department of Pathology, Radboud University Medical Center, 6525 GA Nijmegen, The Netherlands; 2 Department of Immunology, Laboratory Medical Immunology, Erasmus MC, University Medical Center Rotterdam, 3015 CN Rotterdam, The Netherlands; 3 Charité-Universitätsmedizin Berlin, Institute of Pathology, D-10117 Berlin, Germany; 4 Institute of Pathology and Neuropathology, Comprehensive Cancer Center, University Hospital Tübingen, 72076 Tübingen, Germany; 5 Molecular Medicine Program, Central European Institute of Technology, Masaryk University, 62500 Brno, Czech Republic; 6 Department of Hematology, University Hospital Schleswig-Holstein, 24105 Kiel, Germany; 7 Department of Haematology, Belfast City Hospital, Belfast BT9 7AB, UK; 8 Institute of Applied Biosciences, GR 57001 Thermi, Thessaloniki, Greece; 9 Hematology Department, Hospital Pitié-Salpêtrière and Sorbonne University, 75013 Paris, France

**Keywords:** Cancer genetics, Genetics research

## Abstract

One of the hallmarks of B lymphoid malignancies is a B cell clone characterized by a unique footprint of clonal immunoglobulin (IG) gene rearrangements that serves as a diagnostic marker for clonality assessment. The EuroClonality/BIOMED-2 assay is currently the gold standard for analyzing IG heavy chain (IGH*)* and κ light chain (IGK) gene rearrangements of suspected B cell lymphomas. Here, the EuroClonality-NGS Working Group presents a multicentre technical feasibility study of a novel approach involving next-generation sequencing (NGS) of IGH and IGK loci rearrangements that is highly suitable for detecting IG gene rearrangements in frozen and formalin-fixed paraffin-embedded tissue specimens. By employing gene-specific primers for IGH and IGK amplifying smaller amplicon sizes in combination with deep sequencing technology, this NGS-based IG clonality analysis showed robust performance, even in DNA samples of suboptimal DNA integrity, and a high clinical sensitivity for the detection of clonal rearrangements. Bioinformatics analyses of the high-throughput sequencing data with ARResT/Interrogate, a platform developed within the EuroClonality-NGS Working Group, allowed accurate identification of clonotypes in both polyclonal cell populations and monoclonal lymphoproliferative disorders. This multicentre feasibility study is an important step towards implementation of NGS-based clonality assessment in clinical practice, which will eventually improve lymphoma diagnostics.

## Introduction

The vast majority of lymphoid malignancies arise from the unconstrained expansion of a single transformed B or T cell and is characterized by the presence of clonal rearrangements of immunoglobulin (IG) or T cell receptor (TR) genes [1, 2]. In the case of B cell lymphoma, the germline variable (V), diversity (D), and joining (J) genes of the IG loci become rearranged during early development and undergo random deletion and insertion of nucleotides within the junctional regions, thereby generating specific and unique sequences for each B lymphocyte [3]. Additional diversification of the IG repertoire is achieved by the introduction of somatic mutations into the V domain [4]. These “DNA fingerprints” can serve as clonality markers to study lymphoproliferative disorders. Indeed, clonality assessment represents an objective supplementary diagnostic approach to distinguish lymphoid malignancies from non-neoplastic processes [5, 6]. Furthermore, evaluation of the clonal relationship between a primary and recurrent lymphoma via clonality analysis provides an essential tool to confirm the occurrence of a relapse.

At present, the EuroClonality/BIOMED-2 multiplex PCR-based protocol for IG/TR targets combined with heteroduplex analysis or fragment length (GeneScan) analysis, represents the gold standard for clonality assessment of lymphoid tissues [7–9]. Many diagnostic laboratories have extensively validated this protocol for clonality analyses of lymphoma specimens [10–13]. The EuroClonality/BIOMED-2 approach has been designed for optimal DNA samples with amplicons in the range of 150–400 bp. However, in formalin-fixed paraffin-embedded (FFPE) samples with low-quality DNA, clonal rearrangements corresponding to long amplicons may potentially be missed, leading to false-negative results. Moreover, interpretation of clonality patterns in suboptimal DNA samples is not always straightforward, making it difficult to establish whether rearrangement profiles are truly clonal, oligoclonal, or polyclonal. Furthermore, establishing the clonal relationship between a primary lymphoma and its recurrence currently relies mainly on the demonstration of identically sized amplicons; that said, differently sized fragments can still represent the same rearrangement, however they may also describe different clonal lymphoid populations. Confirmation by actual sequence information of the clone is warranted, but Sanger sequencing of less pronounced clones is labor intensive and not always feasible.

In the past decade, high-throughput sequencing of DNA and RNA molecules has been successfully introduced into the field of immunology and hematology, including in-depth analyses of the immune repertoire by next-generation sequencing (NGS)-based detection of IG and TR rearrangements in both physiological and pathological contexts [14–19]. Other applications in hemato-oncology involving V(D)J-NGS include clonal evolution of lymphoid malignancies [20, 21], biomarker tracing employing liquid biopsies [22, 23], and monitoring of minimal residual disease (MRD) [24–27]. The large majority of these studies have been focused on obtaining full sequence coverage of complete IG heavy chain IGHV-IGHD-IGHJ gene rearrangements. In addition, several bioinformatics tools have been developed for V(D)J germline assignment and detection of IGH and IG κ light chain (IGK) rearrangements within reads obtained from NGS datasets [28–32].

In recent years, the EuroClonality-NGS Working Group has developed IG/TR assays, which have been validated across expert European centres to allow for quality-controlled, streamlined, and comprehensive detection of clonal IG/TR rearrangements [33, 34]. Here, we describe the modification and application of the EuroClonality-NGS IGH and IGK assays for B cell clonality detection focusing on small amplicon sizes, highly suitable for analysis of FFPE samples. NGS-based clonality assessment was compared to the conventional EuroClonality/BIOMED-2 approach to evaluate its performance for the detection of IG gene rearrangements.

## Materials and methods

### Tissue specimens

Human peripheral blood and tonsillar tissue were obtained from healthy individuals at Erasmus MC, Rotterdam and Radboudumc, Nijmegen. A total of 14 FFPE tissue specimens from 10 patients with B cell lymphoma were randomly selected for this study (Supplementary Table S1), and analyzed both by conventional EuroClonality/BIOMED-2 assay and NGS. All samples were retrieved from the local archive of the Department of Pathology at Radboudumc and Charité-Universitätsmedizin Berlin and collected in accordance with the declaration of Helsinki.

### Sample preparation for clonality detection by next generation sequencing

Multiplex PCR was adapted from the EuroClonality-NGS IG/TR assays [34], and optimized for B cell clonality detection, including IGHV-IGHD-IGHJ, IGHD-IGHJ, and IGK (IGKV-IGKJ, IGKV-KDE, and Intron RSS-KDE) rearrangements (Supplementary Fig. S1; Supplementary Table S2). EuroClonality-NGS IG primers allow detection of all IGH and IGK genes classified as functional or open-reading frame (ORF) (Supplementary Table S3). Each 25 μl PCR reaction contained 40 ng DNA (or lower amounts in case of sensitivity testing), 2.5 μl GeneAmp 10x PCR Gold Buffer (Thermo Fisher Scientific, Waltham, MA, USA), 0.2 or 0.4 μM of the forward and reverse primers, 200 μM dNTPs, 0.5 U AmpliTaq Gold DNA polymerase (Thermo Fisher Scientific, Waltham, MA, USA), and either 1.5 mM MgCl_2_ (IGHV-VJ-FR3 and IGK primer sets) or 2.0 mM MgCl_2_ (IGH-DJ primer set). After the PCR reactions were completed, the contents of the tubes were pooled and amplicons were purified by the Agencourt AMPure XP kit (Beckman Coulter, Brae, CA, USA).

Details regarding materials and methods, including ion torrent sequencing and bioinformatics analysis are provided as Supplementary Information.

## Results

### IGH and IGK primers for NGS-based clonality assessment

**Fig. 1:**
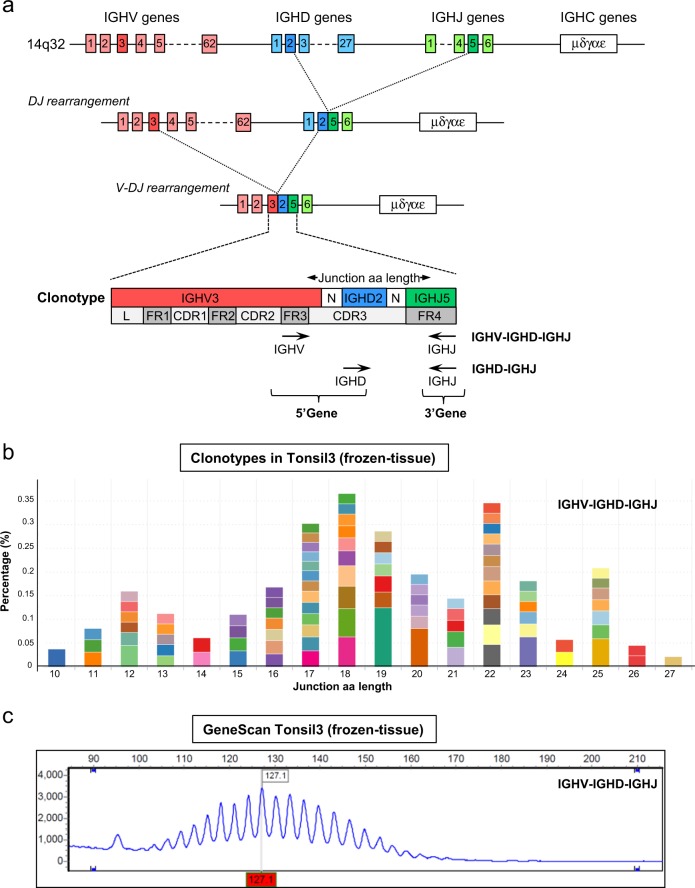
Identification of distinct clonotypes by next generation sequencing (NGS)-based detection of immunoglobulin heavy chain rearrangements. **a** Schematic representation of the immunoglobulin heavy chain (IGH) locus on chromosome 14q32, indicating the presence of 62 IGHV genes, 27 IGHD genes, 6 IGHJ genes, and 5 IGHC genes. Following RAG-mediated DJ and V-DJ rearrangement, each B cell generates one (productive rearrangement) or two (a non-productive and productive rearrangement) specific clonotypes, each consisting of one IGHV, IGHD, and IGHJ gene segment. For the detection of IGHV-IGHD-IGHJ gene rearrangements the forward IGHV primers located in framework region 3 (VH FR3) are combined with IGHJ reverse primers. For the detection of IGHD-IGHJ rearrangements the forward IGHD and reverse IGHJ primers will generate amplicons. Successful amplification will yield DNA fragments that cover the junctional region with a specific amino acid (aa) length, representing the CDR3 region and anchor points. The leader region (L), the conserved framework regions 1–4 (FR1–4) and the hypervariable complementary regions 1–3 (CDR1–3) are indicated. **b** Detection of distinct clonotypes in DNA isolated from a frozen tonsil tissue of a healthy donor (Tonsil3), indicating a polyclonal pattern of IGHV-IGHD-IGHJ rearrangements with variable junction aa length. Each colored bar indicates a distinct clonotype, and the 100 most abundant clonotypes are shown. The NGS data were analyzed and visualized by ARResT/Interrogate. **c** GeneScan profile of Tonsil3 IGHV-IGHD-IGHJ rearrangements as determined with EuroClonality/BIOMED-2 PCR

For the identification of IGHV-IGHD-IGHJ gene rearrangements by NGS, specific forward primers were designed for variable heavy framework 3 (VH FR3) (Fig. 1a), which allows detection of smaller amplicon sizes compared to IGH-VJ-FR1 and IGH-VJ-FR2 forward primers. NGS of IGHV-IGHD-IGHJ gene rearrangements in a control tonsil sample followed by bioinformatics analysis with ARResT/Interrogate [32] resulted in the detection of multiple clonotypes, representing a distribution of different amino acid (aa) junction lengths, which reflected a polyclonal B cell population (Fig. 1b), similar to GeneScan profiles using EuroClonality/BIOMED-2 primers (Fig. 1c). For the identification of partial IGHD-IGHJ gene rearrangements, we employed the same IGH-DJ primer sets as described for MRD marker identification [34]. For the detection of IGK locus gene rearrangements, we also used the same primers as for MRD marker identification, but multiplex PCR was performed within a single tube and PCR conditions were adapted to allow simultaneous amplification of IGKV-IGKJ and IGKV/Intron-KDE gene rearrangements. NGS of IGK locus gene rearrangements in a control tonsil sample yielded a distinctive polyclonal pattern of clonotypes of different aa junction length similar to what could be detected by the EuroClonality/BIOMED-2 profiles for IGKV-IGKJ and IGKV/Intron-KDE gene rearrangements (Supplementary Fig. S2).

### Validation of NGS primers for IGH and IGK clonality analysis

**Fig. 2:**
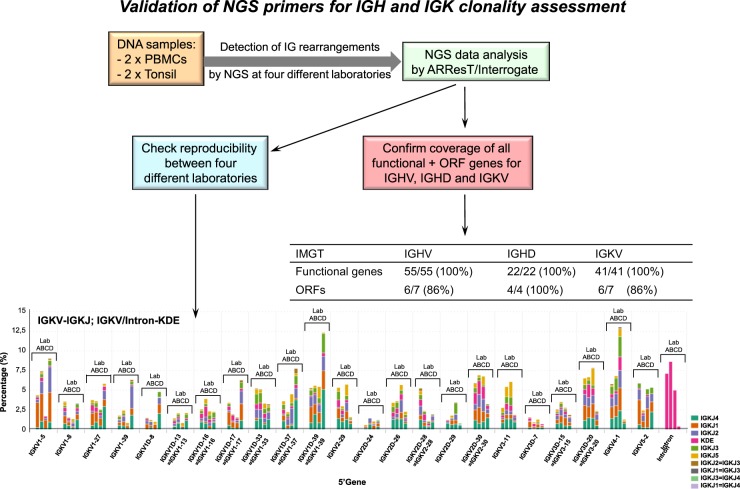
Schematic representation of the procedure to validate the primers designed for immunoglobulin clonality assessment by next-generation sequencing. The lower graph shows a representative plot of the data indicating the relative percentage of the different IGKV genes (5’ gene) in combination with specific IGKJ genes in peripheral blood mononuclear cells (PBMC2). Each set of four bars demonstrate the data as obtained for each of the four centres (Lab A, Lab B, Lab C, and Lab D) participating in the multicentre feasibility study

To validate the IGH and IGK primers using the NGS-based clonality protocol, we performed NGS on different control samples in a multicentre study involving four different centres, namely Radboudumc in Nijmegen (NIJ; Lab A), Erasmus MC in Rotterdam (ROT; Lab B), Charité in Berlin (BER; Lab C), and the Comprehensive Cancer Center in Tübingen (TUB, Lab D). The DNA control samples consisted of two peripheral blood mononuclear cells (PBMC) samples, which are known to harbor 5–15% B cells, and two tonsil tissue specimens derived from healthy donors with ~50–60% B cells. By employing data analysis in ARResT/Interrogate, we examined if all functional and ORF genes for the IGHV, IGHD, and IGKV regions were covered (Fig. 2, Supplementary Table S4). For IGHV, all functional IGHV genes (*n* = 55) and 6 of the 7 ORFs located on chromosome 14q32 were detected by the IGH-VJ-FR3 primer set (Supplementary Table S4). Similar to what has been described for MRD marker identification [34], all 22 functional genes and all 4 ORF of the IGHD region could be identified (Fig. 2). For the IGKV region, all functional genes (*n* = 41), and 6 of the 7 ORFs located on chromosome 2p11 were detected by the IGK-VJ primer set. Thus, virtually all functional IG genes and ORFs were detected by the EuroClonality-NGS primers using the IG clonality protocol.

### Robust detection of IG gene rearrangements in FFPE material by NGS

To assess whether our NGS-based protocol for detection of IG gene rearrangements performed equally well on DNA extracted from frozen and FFPE tissues, we determined the spectrum of IGH and IGK gene rearrangements in both material types, using paired samples of the same biopsy of two independent tonsil specimens. DNA was extracted in one reference laboratory and NGS on amplified IGH and IGK gene rearrangements was performed using Ion Torrent technology in the four different centres (NIJ, ROT, BER, and TUB).

Analysis of the DNA quality showed that the frozen-tissue DNA samples (Tonsil1 and Tonsil3) displayed high DNA quality, while the FFPE samples (Tonsil2 and Tonsil4) displayed shorter fragment length (Supplementary Fig. S3). Bioinformatics analysis by ARResT/Interrogate indicated that, despite the different DNA quality, the variation in total reads obtained for IGH and IGK loci gene rearrangements was largely comparable between frozen and FFPE tissue at each center, ranging from 56,722 to 204,038 reads for frozen-tissue samples, and 40,476 to 224,374 reads for FFPE samples (Supplementary Table S5). The percentage of mapped reads (i.e. reads with an identifiable junction) varied between different centres, ranging from 62% to 88%. The average fraction of mapped reads that could be assigned to IGHV-IGHD-IGHJ gene rearrangements was 37% in frozen-tissue and 28% in FFPE, for IGHD-IGHJ 17% in frozen-tissue and 19% in FFPE, and for IGK locus 46% in frozen-tissue and 54% in FFPE (Supplementary Table S5).

**Fig. 3:**
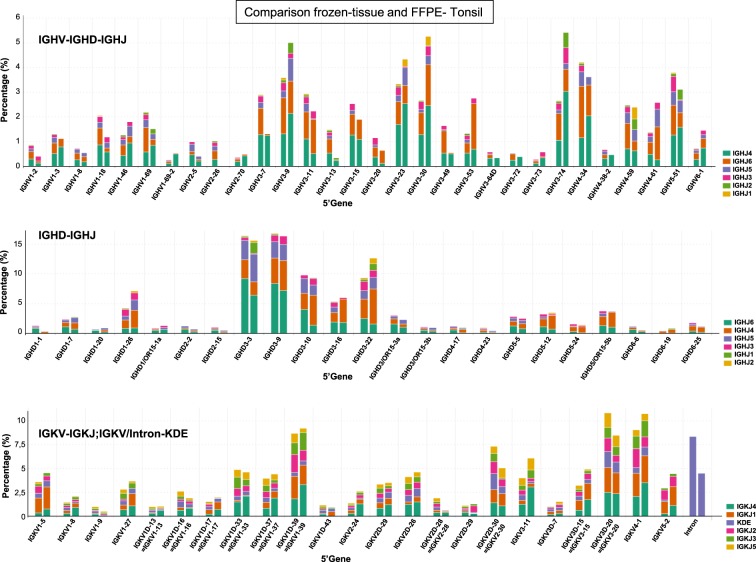
Identification of similar B cell clones in both formalin-fixed and frozen tonsil tissue. IGH (IGHV-IGHD-IGHJ and IGHD-IGHJ) and IGK loci gene rearrangements were sequenced in two DNA samples isolated from either frozen tissue (left bar, Tonsil3) or formalin-fixed paraffin-embedded (FFPE) (right bar, Tonsil4) material derived from the same tonsil specimen. The IGHV, IGHD, and IGKV genes detected are indicated on the *X*-axis (5’ gene) in combination with the different IGHJ (first and second graphs) and IGKJ/KDE genes (third graph) as indicated by the stacked colored bars. Data shown are representative for each of the four centres participating in the multicentre feasibility study

The IGHV, IGHD, and IGKV genes (functional and ORFs) that were identified in the clonotypes of the two tonsil specimens displayed a similar distribution in frozen and FFPE tissue samples in each of the four centres (Fig. 3; Supplementary Table S6). Individual IGHV and IGHD genes were present at higher frequencies in both frozen and FFPE tonsil specimens. The most prevalent IGHV genes included IGHV3-9, IGHV3-23, IGHV3-30, IGHV3-74, and IGHV4-34, while IGHD3-3, IGHD3-9, IGHD3-10, and IGHD3-22 represented the predominant IGHD genes (Fig. 3; Supplementary Table S6). The different IGKV genes were more equally distributed among the rearrangements detected in both frozen and FFPE tissues. Furthermore, we observed that the pattern of IGHJ gene usage for IGHV-IGHJ gene rearrangements was different compared to that for IGHD-IGHJ gene rearrangements (Fig. 3). In both frozen and FFPE tissue, the IGHJ4 gene predominated amongst the IGHV-IGHD-IGHJ rearrangements, while IGHJ6 was more abundant among the IGHD-IGHJ rearrangements. The distribution of IGKJ genes in IGKV-IGKJ gene rearrangements was more balanced, with a slight predominance for the IGKJ4 gene. Collectively, these data demonstrate that there is a high reproducibility in the identification of clonotypes in polyclonal tonsil specimens between frozen and FFPE tissues, and also among different centres.

### Performance parameters of NGS-based clonality analysis

**Fig. 4:**
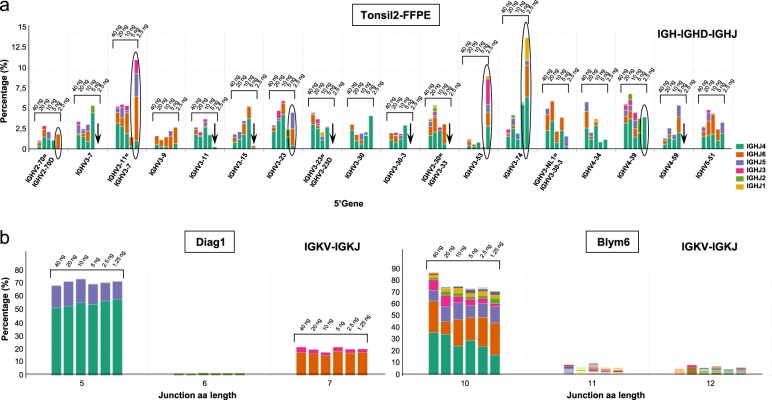
Sensitivity of next-generation sequencing (NGS)-based clonality assessment on lymphoid tissue and lymphoma specimens. **a** The abundance of IG gene rearrangements and the relative distribution of clonotypes was analyzed using decreasing concentrations of Qubit-quantified input DNA for each multiplex PCR reaction, ranging from 40 to 2.5 ng, of a formalin-fixed paraffin-embedded (FFPE) tonsil specimen (Tonsil2). At 2.5 ng input DNA several IGHV-IGHD-IGHJ sequences showed evidence of pseudoclonality (indicated by circles) or were not detected at all (indicated by arrows). **b** Two clonal B cell lymphoma specimens (Diag1 and Blym6) were tested in NGS-based clonality analysis using decreasing amount of input DNA (40–1.25 ng). The data as obtained for IGKV-IGKJ gene rearrangement analysis is presented, with detection of the abundant clone even at 1.25 ng input DNA. The green (IGKV5-2/IGKJ2) and orange (IGKV1D-8/IGKJ4) bar of Diag1 represent bi-allelic IGKV-IGKJ gene rearrangements. The green (IGKV1(D)-39/IGKJ2 = J3) and orange (IGKV1(D)-39/IGKJ2) bars of Blym6 represent the same clonotype but ARRest/Interrogate distinguished two variants, since the sequence of the IGKJ gene was assigned differently. The purple, pink, and other colored bars represent additional minor clonotypes

Next, the minimal DNA amount that would be required for reliable performance of clonality assessment was determined. This is crucial information for the interpretation of clonality in case of tumor specimens with scarce presence of lymphoid cells or small biopsies. A known pitfall in these situations is the risk of pseudoclonality, which may arise from selective amplification of only a few target cells [35]. By analyzing the pattern of IGH and IGK locus gene rearrangements with decreasing concentrations of input DNA isolated from polyclonal tonsil tissue of FFPE origin, the transition from a regular polyclonal rearrangement pattern to pseudoclonality or absence of IG gene rearrangement was determined. Analysis of IGHV-IGHD-IGHJ gene rearrangements demonstrated that 40, 20, and 10 ng of input DNA for each multiplex PCR reaction produced a similar distribution and abundance of IGHV and IGHJ genes (Fig. 4a). At 5 ng DNA, the polyclonal profile started to change to a less diverse rearrangement pattern, while at 2.5 ng DNA there was clear evidence of pseudoclonality or immunodominance of several IGHV genes, with many other IGHV genes not being detected (Fig. 4a). These analyses indicate that an input range of 10–40 ng of Qubit quantified DNA per multiplex PCR can be used for IG clonality analysis. Nevertheless, a larger series of small tissue biopsies with variable amounts of suspect malignant lymphocytes and reactive lesions need to be analyzed to assess the exact minimum amount for reliable clonality assessment in biopsy material.

In a similar approach, we also investigated the limit of detection using two clonal B cell lymphoma specimens (Diag1: 80% tumor cells; Blym6: 10% tumor cells), by testing a range of 1.25–40 ng of input DNA for each multiplex PCR reaction. Here, clonal rearrangements could still be detected at the lowest input concentration of 1.25 ng (Fig. 4b), indicating that NGS can detect rearrangements at this low DNA concentration in case of a clonal B cell lymphoma. Next, we analyzed four B cell lymphoma specimens (Blym1, Blym3, Blym5, and Diag1) with a high tumor load (80–90%) in a dilution series at concentrations of 10%, 5%, 2.5%, and 1% in a polyclonal background of FFPE tonsil DNA, using a fixed amount of total input DNA (40 ng). The data for IGHV-IGHD-IGHJ, IGHD-IGHJ, and IGKV-IGKJ gene rearrangements demonstrated that, in the majority of cases, clonal rearrangements could be traced back at 10%, 5%, and 2.5% dilutions (Supplementary Fig. S4). For two of the four B cell lymphoma dilution series, the abundant clonotype for IGHD-IGHJ and IGKV-IGKJ gene rearrangements could even be detected at a level of 1% within the polyclonal background signal (Supplementary Fig. S4).

### Comparison between EuroClonality/BIOMED-2 and NGS for clonality assessment

**Fig. 5:**
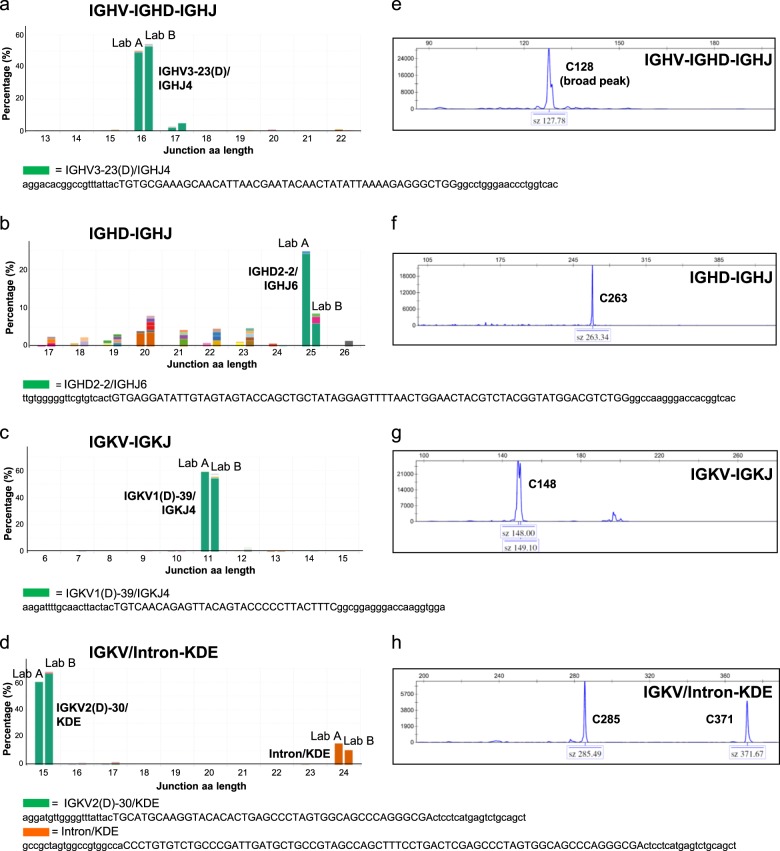
Next-generation sequencing (NGS)-based clonality assessment detects clonal rearrangements in B cell lymphoma specimen. B cell lymphoma specimen Blym1 was analyzed for IGH (IGHV-IGHD-IGHJ and IGHD-IGHJ) and IGK (IGKV-IGKJ and IGKV/Intron-KDE) loci gene rearrangements by NGS (**a–d**) in two independent centres (Lab A and Lab B) and compared to EuroClonality/BIOMED-2 assay (**e**, **f**). The sequence of the abundant clonotypes is shown, with the nucleotide junction sequence in capitals [33], and flanking sequences in lower case, which have been trimmed to 20 nucleotides length

Prior to implementation of NGS-based analysis of IG gene rearrangements for clonality detection of lymphoid malignancies, we wanted to critically compare this novel technique to the gold standard EuroClonality/BIOMED-2 assay. To this end, a total of 14 distinct B cell lymphoma samples that were previously tested with EuroClonality/BIOMED-2 PCR and GeneScan analysis in routine diagnostics, were included in our NGS multicentre study (Supplementary Table S1; Supplementary Fig. S5). The IGHV-IGHD-IGHJ, IGHD-IGHJ, IGKV-IGKJ, and IGKV/Intron-KDE clonotypes were determined by NGS in two different centres and analyzed by ARResT/Interrogate. The graphical output of the clonotypes as obtained by NGS was compared with the EuroClonality/BIOMED-2 GeneScan data. In the majority of cases (38 out of 50 targets with a clonal rearrangement), the number of dominant IG gene rearrangements that could be identified by NGS was identical to those identified using the EuroClonality/BIOMED-2 approach (Table 1; Supplementary Table S7). For instance, in B cell lymphoma specimen Blym1, abundant clonotypes for IGHV-IGHD-IGHJ (IGHV3-23(D)/IGHJ4), IGHD-IGHJ (IGHD2-2/IGHJ6), IGKV-IGKJ (IGKV1(D)-39/IGKJ4), IGKV-KDE (IGKV2(D)-30/KDE), and Intron-KDE were detected, although in this sample the results for IGHD-IGHJ gene rearrangement detection slightly varied between the two centres (Fig. 5).

**Table 1:** Summary immunoglobulin clonality assessment by GeneScan-EuroClonality/BIOMED-2 assay and next-generation sequencing (NGS)

	IGHV-IGHD-IGHJ GeneScan NGS		IGHD-IGHJ GeneScan NGS		IGKV-IGKJ GeneScan NGS		IGKV/Intron-KDE GeneScan NGS	
Blym1	1R	1R	1R	1R	1R	1R	2R	2R
Blym2	1R+PCB	1R^a^	1R	1R	1R+PCB	2R	2R+PCB	2R
Blym3	1R	O^b^	1R	1R	1R+PCB	2R	2R	2R
Blym4	1R	1R	1R+PCB	1R	1R+PCB	2R	1R	1R
Blym5	O	1R	1R	1R	1R	1R	2R	2R
Blym6	1R	1R	Nsp	1R^a^	1R+PCB	1R	1R	1R
Diag1	1R	1R	1R	1R	1R	2R	2R	2R
Relap1	1R	1R	1R	1R	1R	2R	2R	2R
Diag2	Nsp	O	1R	1R	2R	2R	2R	2R
Relap2	1R	1R	1R	1R	1R	1R	1R+PCB	2R
Diag3	P	P	P	P	P	P	1R	1R
Relap3	P	P	1R+PCB	1R+PCB	P	1R+PCB	1R+PCB	1R
Diag4	P	1R	1R	1R	Nsp	P	Nsp	2R
Relap4	1R+PCB	1R+PCB	1R+PCB	1R	1R+PCB	1R+PCB	1R+PCB	1R+PCB

Colors used: blue, inferior performance NGS compared to GeneScan with EuroClonality/BIOMED-2 primers; green, improved performance NGS compared to GeneScan with EuroClonality/BIOMED-2 primers

*1R* one clonal gene rearrangement, *2R* two clonal gene rearrangements, *PCB* polyclonal background, *O* oligoclonal: multiple clones, *Nsp* no specific product, *P* polyclonal

^a^Related clonotypes belonging most likely to one gene rearrangement, single nucleotide variations probably due to somatic hypermutation or sequencing artifacts

^b^Mismatches on template of NGS IGHV primer probably due to somatic hypermutation

**Fig. 6:**
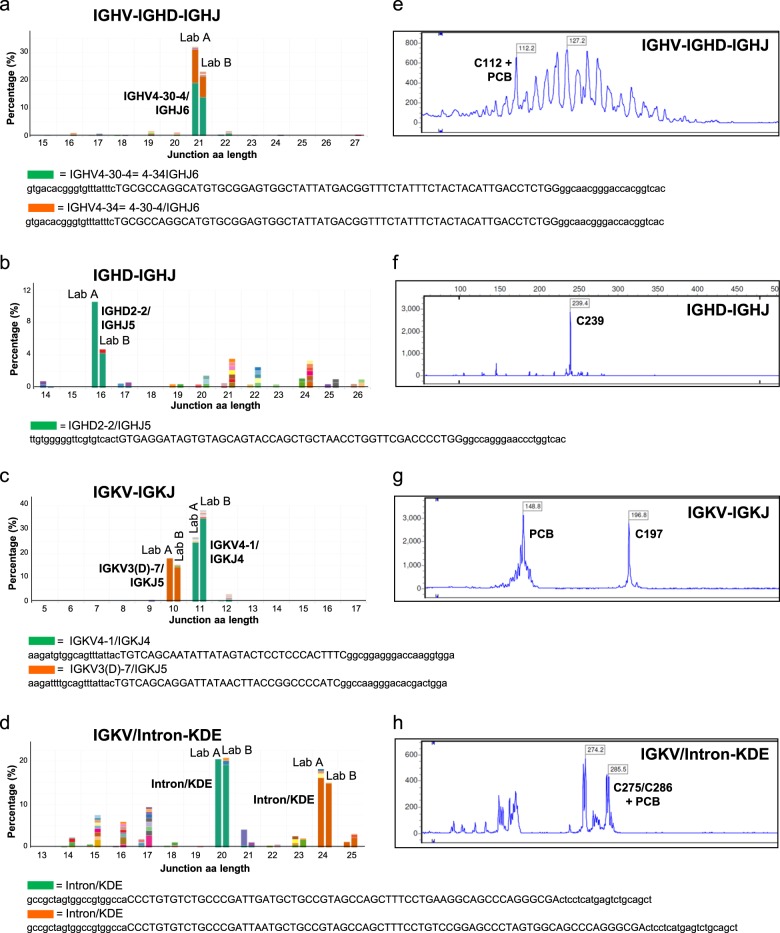
Detection of immunoglobulin gene rearrangements in lymphoma specimen with low tumor load by next-generation sequencing (NGS)-based clonality assessment. B cell lymphoma specimen Blym2 (20% malignant B cells) was analyzed for IGH (IGHV-IGHD-IGHJ, IGHD-IGHJ) and IGK (IGKV-IGKVJ and IGKV/Intron-KDE) loci gene rearrangements by NGS (**a**–**d**) in two independent laboratories (Lab A and Lab B) and compared to EuroClonality/BIOMED-2 assay (**e**, **f**). The sequence of the abundant clonotypes is shown, with the nucleotide junction sequence in capitals [33], and flanking sequences in lower case, which have been trimmed to 20 nucleotides length. PCB polyclonal background

Importantly, for 11 out of 50 targets (22%), NGS-based clonality assessment identified clonal rearrangements that were not detected by EuroClonality/BIOMED-2, an improvement primarily due to the alternative primer design towards shorter amplicons (Table 1). For instance, in Blym2, the EuroClonality/BIOMED-2 primers identified only one abundant IGKV-IGKJ gene rearrangement, while NGS detected the presence of bi-allelic IGKV-IGKJ gene rearrangements with the concomitant identification of two clonotypes (IGKV4-1/IGKJ4 and IGKV3(D)-7/IGKJ5) (Fig. 6). The IGKV4-1/IGKJ4 clonal rearrangement is expected to yield a product in the 275–295 bp range using the EuroClonality/BIOMED-2 approach, which could not be amplified in this apparently suboptimal DNA sample. In Blym4, NGS again detected bi-allelic IGKV-IGKJ gene rearrangements, of which only one was detected by the EuroClonality/BIOMED-2 assay (Supplementary Fig. S6). There was only one instance in which the NGS-based approach failed to detect a rearrangement that was identified with EuroClonality/BIOMED-2 primers (Table 1; Supplementary Table S8); in Blym3, the dominant IGHV-IGHD-IGHJ gene rearrangement detected by GeneScan, was only present as a minor clonotype using NGS, which could be attributed to two mismatches between the DNA sequence and the NGS IGHV primer. Collectively, these results demonstrate that NGS-based detection of IG gene rearrangements is a feasible approach for clonality assessment of FFPE specimens of lymphoproliferative disorders, and even shows improved performance compared with the current EuroClonality/BIOMED-2 approach.

### Analysis of diagnosis-relapse cases by NGS-based detection of IG gene rearrangements

**Fig. 7:**
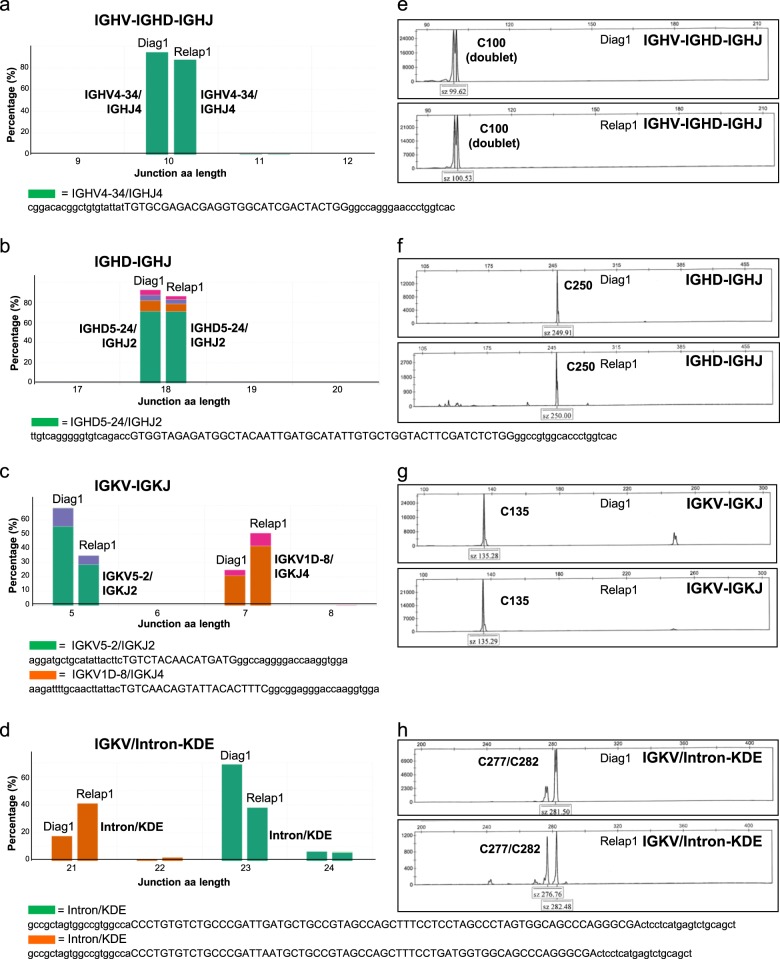
Comparison paired diagnosis-relapse B cell lymphoma specimen by next-generation sequencing (NGS)-based clonality analysis. Two consecutive B cell lymphoma specimens (Diag1 and Relap1) from the same patient were analyzed for IGH (IGHV-IGHD-IGHJ and IGHD-IGHJ) and IGK (IGKV-IGKJ and IGKV/Intron-KDE) loci gene rearrangements by NGS (**a–d**) and compared to EuroClonality/BIOMED-2 assay (**e**, **f**). The sequence of the abundant clonotypes are shown, with the nucleotide junction sequence in capitals [33], and flanking sequences in lower case, which have been trimmed to 20 nucleotides length

At present, the clonal relationship between B cell lymphoma recurrences at subsequent timepoints or from multiple lesions at different locations is mostly established by fragment length analysis of IG gene rearrangements using the EuroClonality/BIOMED-2 assay. However, in some situations these assays remain inconclusive, because the limited amounts of biopsy material or archival tissue material yielding suboptimal DNA quality, does not allow perfect reproducibility of the obtained GeneScan or heteroduplex patterns. NGS-based clonality assessment has the advantage of generating sequence information of the detected clonotypes, thereby providing definite evidence whether tumors are clonally related or not. In our multicentre validation study we included four paired diagnosis-relapse samples (Supplementary Table S1) to assess the added value of the NGS-based approach for this application. In the Diag1 and Relap1 samples, a single IGHV-IGHD-IGHJ and IGHD-IGHJ gene rearrangement could be detected, which showed identical clonotypes and sequence information for the paired diagnosis and relapse samples (Fig. 7). For IGK, two clonotypes could be identified for IGKV-IGKJ and IGKV/Intron-KDE, respectively, in both diagnosis and relapse samples, which harbored identical sequences in both lymphoma specimens, thereby establishing their clonal relationship.

On the other hand, the distinct clonotypes that were identified for IGH and IGK gene rearrangements in the paired Diag2 and Relap2 samples confirmed the data from the GeneScan analysis that these were clonally unrelated B cell tumors (Supplementary Table S1 and S7). In samples Diag3 and Relap3, a clonal IGKV-KDE rearrangement of the same clonotype could be identified in each of the samples, thus providing evidence that these two samples were most likely clonally related. Finally, the paired samples Diag4 and Relap4 displayed distinct IGHV-IGHD-IGHJ and IGHD-IGHJ gene rearrangements at diagnosis and relapse, while specific IGK locus rearrangements were only detected at diagnosis (Supplementary Table S7), suggesting that these lymphoma samples were most likely clonally distinct. In conclusion, the NGS-based approach for clonality assessment provides the desired diagnostic accuracy in confirming the clonal relationship of the amplified rearrangements in multiple lesions over time.

## Discussion

Clonality testing for IGH and IGK locus gene rearrangements is an important diagnostic test to distinguish reactive lesions from malignant lymphoproliferative disorders and to establish the clonal relationship of recurrent lesions within the same patient. With the advent of high-throughput NGS of B cell receptor V(D)J gene rearrangements and the development of bioinformatics platforms for processing analysis and visualization, a more sensitive approach for clonality assessment is within reach. As the EuroClonality-NGS Working Group, we here present a multicentre feasibility study employing deep sequencing of rearranged IGH and IGK genes to analyze the clonal composition of B cell lymphoma samples, also suitable for DNA isolated from FFPE specimens. Tumor-specific IGK locus gene rearrangements and their specific clonotypes could be detected in all 14 samples (100%) analyzed by NGS, while IGHV-IGHD-IGHJ and IGHD-IGHJ gene rearrangements were detected in 10 of 14 (71%), and 13 of 14 (93%) B cell lymphoma cases by NGS, respectively. Overall, a specific clonotype could be detected by NGS in all 14 samples analyzed. Even though this is a high fraction, larger validation studies are required to more firmly establish the high clinical sensitivity of our novel EuroClonality-NGS B cell clonality approach.

We established that for clonality assessment an input range of 10-40 ng of Qubit-quantified DNA is required for each multiplex PCR reaction, while in a lymphoma specimen with a high percentage of suspect malignant lymphoid cells, even 5 ng DNA might allow detection of a clonotype. However, the absolute minimum amount of DNA for reliable clonality analysis, especially from small biopsies with a much more restricted B cell repertoire, still needs to be assessed in future studies using a larger series of such biopsies.

Similar to the BIOMED-2 approach, NGS-based clonality assessment also relies on an initial multiplex PCR step, which could still be hampered by somatic hypermutation and specific polymorphisms that might prevent primer annealing. Nonetheless, we observed that our newly designed primers, which are aimed to generate shorter amplicons suitable for NGS-based clonality assessment on both Ion Torrent and Illumina platforms, allowed identification of additional rearrangements that could not be detected by the conventional EuroClonality/BIOMED-2 assay. From the 14 lymphoma specimens that were analyzed, NGS could additionally detect 12 clonotypes (two IGHV-IGHD-IGHJ, one IGHD-IGHJ, six IGKV-IGKJ and three IGKV/Intron-KDE) compared to the EuroClonality/BIOMED-2 approach. Conversely, the NGS approach failed to detect one abundant clonotype that was detected with the EuroClonality/BIOMED-2 approach, probably due to mismatches within the NGS forward primer. Furthermore, when we analyzed the paired diagnosis–relapse samples we observed that the available sequence information of the specific clonotypes provided supportive and detailed information for establishing clonal relationship of the B cell lymphoma recurrences.

We demonstrated that all functional IGHV, IGHD, and IGKV genes, as well as the majority of the ORF genes were detectable by the EuroClonality-NGS approach. However, we observed that the relative representation of the individual IG genes varied to some extent between the different centres. This variation seemed to partially correlate with distinct efficiencies of the individual multiplex PCR and the assignment of mapped reads due to differences in local facilities and Ion Torrent sequencers (Supplementary Table S5). Thus, internal controls employing defined and standardized polyclonal samples, or established spike-in standards as described elsewhere [33], will be important quality controls for NGS-based approach of IG clonality assessment in a diagnostic setting.

In the current study, we observed distinct usage of the different V and J genes in the B lymphocyte populations of peripheral blood and tonsil control samples of healthy individuals. The IGHV3 subgroup genes were the most frequent, with a strong predominance of IGHV3-7, IGHV3-21, IGHV3-23, IGHV3-30, and IGHV3-48 genes (Supplementary Table S4), similar to what others have described in repertoire analyses in normal controls [14, 15]. Furthermore, the IGHJ4 gene predominated in IGHV-IGHD-IGHJ gene rearrangements, in keeping with observations in naïve, transitional, and memory B cells [15]. For IGHD genes, there was bias to the usage of the IGHD3-3, IGHD3-9, IGHD3-10, and IGHD3-22 genes, as also detected by others [14]. Overall, these findings demonstrate that our newly designed and validated NGS primers can be applied for several high-throughput immunogenetic applications, more particularly clonality assessment, as indicated in this report, and MRD marker identification [34]. A comparison in turnaround time and costs between conventional EuroClonality/BIOMED-2 and NGS is described in Supplementary Fig. S7 and Supplementary Table S9. By extending these studies to various patient categories and immunological entities with a predisposition to lymphoid malignancies, such as Sjögren syndrome, and lymphoproliferative disorders associated with viral and bacterial infections, specific guidelines should eventually be defined to distinguish reactive lesions from malignant lymphoma.

Indeed, an important aspect that has to be re-evaluated in the context of NGS-based clonality assessment is the term clonality and its clinical usage. In particular, in lymphoma specimens with low numbers of (suspect) malignant lymphoid cells, few reads from malignant B cell clones might be indistinguishable from reactive B cell clonotypes, especially in those cases without knowledge of an already identified B cell clone. Therefore, it should be firmly established which repertoire is to be expected in healthy individuals, and in patients with reactive clonal expansions due to infections, or those with immunological disorders. Moreover, future studies need to define the spectrum of mono-, oligo-, and polyclonality in patient samples, and also recognize certain patterns of repertoire skewing that may indicate reactive lesions, thereby defining clinically relevant thresholds for clonality assessment.

## Supplementary information

Supplementary information

Supplementary Table S9
