# Supplementary material for: Next-generation sequencing of immunoglobulin gene rearrangements for clonality assessment: a technical feasibility study by EuroClonality-NGS
Source: Leukemia. 2019 Jun 13;33(9):2227–40. doi: 10.1038/s41375-019-0508-7 (PMC6756030; doi:10.1038/s41375-019-0508-7)
Supplement: Supplementary file 1 — Supplementary information [file 41375_2019_508_MOESM1_ESM.pdf]

Next-generation sequencing of immunoglobulin gene rearrangements for clonality assessment: a technical feasibility study by EuroClonality-NGS

Blanca Scheijen, Ruud WJ Meijers, Jos Rijntjes, Michèle Y van der Klift, Markus Möbs, Julia Steinhilber, Tomas Reigl, Michiel van den Brand, Michaela Kotrova, Julia-Marie Ritter, Mark A Catherwood, Kostas Stamatopoulos, Monika Brüggemann, Frédéric Davi, Nikos Darzentas, Christiane Pott, Falko Fend, Michael Hummel, Anton W Langerak and Patricia JTA Groenen, on behalf of the EuroClonality-NGS Working Group.

SUPPLEMENTARY INFORMATION

This file contains Supplementary Materials and Methods, Supplementary Tables, Supplementary Figures and Supplementary Figure Legends. Supplementary Table S9 is an Excel file.

SUPPLEMENTARY MATERIALS AND METHODS

DNA isolation

DNA was isolated with the classical DNA extraction method from FFPE tissue involving xylene and ethanol washing steps followed by incubation in lysis buffer in the presence of proteinase K and Chelex beads. Subsequently, DNA was purified using commercial QIAamp DNA Mini Kit (Qiagen, Venlo, The Netherlands). The concentration of DNA was determined by the Qubit® 2.0 fluorometer according to the manufacturers' instruction (Invitrogen, Carlsbad, CA, USA). The quality of the DNA was determined on an Agilent Genomic DNA ScreenTape using the Agilent 2200 TapeStation system (Agilent, Santa Clara, CA, USA), KAPA SYBR FAST Universal qPCR Master Mix (KAPABiosystems, Wilmington, MA, USA) according to the manufacturer's instruction, or a quality control PCR generating discrete PCR amplicons (100 bp, 200 bp, 300 bp and 400 bp fragments) as described in : Dongen JJM, Langerak AW, Brüggemann M, Evans PAS, Hummel M, Lavender FL *et al*. Design and standardization of PCR primers and protocols for detection of clonal immunoglobulin and T-cell receptor gene recombinations in suspect lymphoproliferations: Report of the BIOMED-2 Concerted action BMH4-CT98-3936. *Leukemia* 2003 Dec 12; **17**(12): 2257-2317).

EuroClonality/BIOMED-2 assay and detection of amplification products

Conventional B cell clonality was assessed using the EuroClonality/BIOMED-2 master mixes for IGH framework 1 (FR1), FR2, FR3 (tubes A-C), IGH DJ tube A, and IGK (tubes A and B) using ABI

Fluorescence Detection (InVivoScribe Technologies, San Diego, CA, USA) according to the manufacturer's instructions. The fluorescently labeled PCR products were detected by capillary gel electrophoresis using the ABI 3500 Genetic Analyzer (Applied Biosystems, Thermo Fisher Scientific, Waltham, MA, USA) and analyzed by fragment analysis using GeneScan^{TM} 600LIZ Size Standard v2.0 (Applied Biosystems, Thermo Fisher Scientific, Waltham, CA, USA), which discriminates amplification products according to their size.

Next generation sequencing by Ion Torrent

The Ion Plus Fragment Library Kit (Thermo Fisher Scientific, Waltham, CA) was used to end-repair the amplicons (40 ng), to ligate the adaptors for barcoding each library (Ion Xpress Barcode Adaptors, Thermo Fisher Scientific, Waltham, CA), for the nick repair and for the amplification of the library. The DNA concentration for each library was determined with Qubit Fluorimetric Quantification (Thermo Fisher Scientific, Waltham, CA) before pooling the libraries at equivalent DNA quantities. Each of the four labs that participated in the multicentre study to validate the assay performed multiplex PCR on 21 samples in total, which in the end were diluted to a final concentration of 10-20 ng/ml of pooled libraries. These 21 samples were loaded on an Ion Torrent 318- or 520-chip and sequenced following a standardized procedure of the local Sequence Facility (Ion PGM template OT2 200 kit, Ion Chef, Ion OneTouch 2 or Ion S5 XL system; Thermo Fisher Scientific, Waltham, CA). The standard operating procedure can be found on www.euroclonality.org/protocols. Data has been deposited at EMBL/EBI European Nucleotide Archive (ENA), accession code PRJEB32203.

Bioinformatics analysis

The sequence data sets were analyzed and visualized using ARResT/Interrogate (version 0.25.001). For data analysis and visualization the combination of two feature types was used, i.e. either "5' gene" and "3' gene" for a more abstract view, or "junction amino acid (aa) length" and "clonotype" for a detailed spectratyping-view. A clonotype is defined by the 5' and 3' gene annotation, the segmentation and aa sequence of the junction based on strict pre-clustering to accommodate minor less-abundant sequence variations, and the predicted functionality of the rearrangement (for more details see accompanying manuscript by Knecht et al, this issue).

SUPPLEMENTARY TABLES

Supplementary Table S1

Characteristics B cell lymphoma specimens

B cell lymphoma	Tumor type at diagnosis* (Internal code)	Fraction B cells in specimen#	Percentage suspected tumor cells	DNA quality† (PCR)	BIOMED-2 IGHV-IGHD-IGHJ GeneScan	BIOMED-2 IGHD-IGHJ GeneScan	BIOMED-2 IGKV-IGKJ GeneScan	BIOMED-2 IGKV/Intron-KDE GeneScan
Blym1	DLBCL abdomen (DPA12-926)	80%	100%	300w	C128 (broad peak)	C263	C148	C285/C371
Blym2	EBV+ PTLD (DPA16-294)	30%	70%	200	C112 + PCB	C239	C197 + PCB	C274/C286 + PCB
Blym3	Cutaneous DLBCL (DPA13-1063)	90%	90%	300w	C121	C262	C291 + PCB	C278/C284
Blym4	Gastrointestinal MZL (Berlin1082-15)	25%	80%	300	C130	C135 + PCB	C197 + PCB	C282
Blym5	Anaplastic plasmacytoma; extraosseous (Berlin1325-15)	80%	>90%	300	Oligoclonal	C258	C131$/ C181	C241 + weak C279
Blym6	EBV+ PTLD (DPA14-1568)	10%	90%	400w	C139	No specific product	C145 + PCB	C285
Diag1	Uterine cervix DLBCL (DPA12-726)	80%	100%	400	C100 (doublet)	C250	C135	C277/C282
Relap1	Vermiform appendix DLBCL (DPA12-728)	80%	100%	300	C100 (doublet)	C250	C135	C277/C282
Diag2	Testicular DLBCL (DPA13-689)	80%	100%	300	No specific product	C251	C146/ C195	C276/C281
Relap2	CLL/SLL bone marrow (DPA13-690)	30%	90%	400	C121	C149	C282	C242 + PCB
Diag3	Nodal cervical MZL (DPA14-1349)	50%	90%	400	Polyclonal	Polyclonal	Polyclonal	C234
Relap3	Nodal cervical MZL (DPA15-208)	60%	100%	400	Polyclonal	Weak C138 + PCB	Polyclonal	C234 + PCB
Diag4	Glandula parotis DLBCL (DPA17-891)	50%	90%	100	Polyclonal	C233	No specific product	No specific product
Relap4	Extranodal lung MZL (DPA17-892)	20%	90%	300	Weak C117 + PCB	C259 + PCB	Weak C198 + PCB	Weak C230 + PCB

* Abbreviations used: DLBCL, Diffuse large B cell lymphoma; EBV, Epstein-Barr virus; MZL, marginal zone lymphoma; PTLD, post-transplant lymphoproliferative disorder; CLL, chronic lymphocytic leukemia; SLL, small lymphocytic lymphoma; PCB, polyclonal background band.

# The fraction B lymphoma cells is the percentage of suspected tumor cells within the population of B cells present in the FFPE specimen as determined by H&E morphology and immunohistochemistry for pan-B cell markers.

† Readout of the quality control PCR with standardized fragment lengths (100 bp, 200 bp, 300 bp and 400 bp); w, weak intensity.

$ The C131 bp fragment results from mispriming of the Vk1f/6 forward primer and represent the same R as C181.

Supplementary Table S2

Primers included in multiplex PCR reaction for NGS-based clonality assessment

Tube IGHV	Final concentration	Primer Sequence
IGH-V-FR3-A-1	0.4 µM	AAGTTCCAGGGCAGAGTCAC
IGH-V-FR3-B-1	0.4 µM	GTCCATCAGCACAGCCTACA
IGH-V-FR3-C-1	0.4 µM	GACATGTCCACAAGCACAGC
IGH-V-FR3-D-1	0.2 µM	TCTCCAAGGACACCTCCAAGA
IGH-V-FR3-E-1	0.2 µM	CAGGCTCACCATCTCCAAGG
IGH-V-FR3-F-1	0.2 µM	CCATCTCTGAAGAGCAGGCT
IGH-V-FR3-G-1	0.4 µM	TGAAGGGCCGATTCACCATC
IGH-V-FR3-H-1	0.4 µM	AGGCAGATTCACCATCTCAAGA
IGH-V-FR3-I-1	0.4 µM	AGCGCCGATTCATCATCTCC
IGH-V-FR3-J-1	0.2 µM	CCAAAAGCATCACCTATCTGCA
IGH-V-FR3-K-1	0.2 µM	GAAGGGCCGGTTCACCATC
IGH-V-FR3-L-1	0.2 µM	ACCTCCAGAGATAACGCCAAG
IGH-V-FR3-M-1	0.2 µM	CAGGAAGGGCAGATTCACCA
IGH-V-FR3-N-1	0.2 µM	GAAGGGCCGATTGACCATCTC
IGH-V-FR3-O-1	0.4 µM	CTCCGTGAAGGGCAGATTCA
IGH-V-FR3-P-1	0.2 µM	GATGATTCAAAGAACACGGCGT
IGH-V-FR3-Q-1	0.4 µM	CCGTCCCTCAAGAGTCGAGT
IGH-V-FR3-R-1	0.4 µM	CCGTCCCTCAAGAGTCGAAT
IGH-V-FR3-S-1	0.2 µM	GTCACCATCTCAGCCGACAA
IGH-V-FR3-T-1	0.2 µM	CAAGTCCATCAGCACTGCCT
IGH-V-FR3-U-1	0.4 µM	CAGTTCTCCCTGCAGCTGAA
IGH-V-FR3-V-1	0.4 µM	GGCTTCACAGGACGGTTTGT
IGH-J-A-1	0.2 µM	CTTACCTGAGGAGACGGTGACC
IGH-J-B-1	0.2 µM	CTCACCTGAGGAGACAGTGACC
IGH-J-C-1	0.2 µM	CTCACCTGAGGAGACGGTGACC

Tube IGHD	Final concentration	Primer Sequence
IGH-D-A-1	0.2 µM	GATTCYGAACAGCCCCGAGTCA
IGH-D-B-1	0.2 µM	GATTTTGTGGGGGYTCGTGTC
IGH-D-C-1	0.2 µM	GTTTGRRGTGAGGTCTGTGTCA
IGH-D-D-1	0.2 µM	GTTTRGRRTGAGGTCTGTGTCACT
IGH-D-E-1	0.2 µM	CTTTTTGTGAAGGSCCCTCCTR
IGH-D-F-1	0.2 µM	GTTATTGTCAGGSGRTGTCAGAC
IGH-D-G-1	0.2 µM	GTTATTGTCAGGGGGTGYCAGRC
IGH-D-H-1	0.2 µM	GTTTCTGAAGSTGTCTGTRTCAC
IGH-J-A-1	0.2 µM	CTTACCTGAGGAGACGGTGACC
IGH-J-B-1	0.2 µM	CTCACCTGAGGAGACAGTGACC
IGH-J-C-1	0.2 µM	CTCACCTGAGGAGACGGTGACC

Tube IGK	Final concentration	Primer Sequence
IGK-V-A-1	0.2 µM	AAGTGGGGTCCCATCAAGGTTCAG
IGK-V-B-1	0.2 µM	AGTCCCATCTCGGTTCAGTGGCAG
IGK-V-C-1	0.2 µM	GAAACAGGGGTCCCATCAAGGTTC
IGK-V-D-1	0.2 µM	TCCCAGACAGATTCAGTGGCAGTG
IGK-V-E-1	0.2 µM	CTGGAGTGCCAGATAGGTTCAGTG
IGK-V-F-1	0.2 µM	CCCTGGAGTCCCAGACAGGTTCAG
IGK-V-G-1	0.2 µM	GCATCCCAGCCAGGTTCAGTG
IGK-V-H-1	0.2 µM	GTCCCTGACCGATTCAGTGGCA
IGK-V-I-1	0.2 µM	AATCCCACCTCGATTCAGTGGC
IGK-V-J-1	0.2 µM	CTCAGGGGTCCCCTCGAGGTT
IGK-V-K-1	0.2 µM	AGACACTGGGGTCCCAGCCA
IGK-INTR-A-1	0.2 µM	GAGTGGCTTTGGTGGCCATGC
IGK-DE-A-1	0.2 µM	GCAGCTGCAGACTCATGAGGAG
IGK-J-A-1	0.2 µM	ACGTTTGATCTCCACCTTGGTCCC
IGK-J-B-1	0.2 µM	ACGTTTGATATCCACTTTGGTCCC
IGK-J-C-1	0.2 µM	ACGTTTAATCTCCAGTCGTGTCCC

Supplementary Table S3

Human IGHV genes (IMGT database)

	**Functional gene (F)***		**Open reading frame (ORF)[#]**		**Pseudogene (P)[†]**	
1	IGHV1-2	14q32.33	IGHV1-38-4	14q32.33	IGHV1-12	14q32.33
2	IGHV1-3	14q32.33	IGHV1/OR15-1	15q11.2	IGHV1-14	14q32.33
3	IGHV1-8	14q32.33	IGHV1/OR15-5	15q11.2	IGHV1-17	14q32.33
4	IGHV1-18	14q32.33	IGHV1/OR15-9	15q11.2	IGHV1-67	14q32.33
5	IGHV1-24	14q32.33	IGHV1/OR21-1	21p11.2	IGHV1-68	14q32.33
6	IGHV1-45	14q32.33	IGHV2/OR16-5	16p11.2	IGHV1-NL1	14q32.33
7	IGHV1-46	14q32.33	IGHV3-16	14q32.33	IGHV1/OR15-2	15q11.2
8	IGHV1-58	14q32.33	IGHV3-25	14q32.33	IGHV1/OR15-3	15q11.2
9	IGHV1-69	14q32.33	IGHV3-35	14q32.33	IGHV1/OR15-4	15q11.2
10	IGHV1-69D	14q32.33	IGHV3-38	14q32.33	IGHV1/OR15-6	15q11.2
11	IGHV1-69-2	14q32.33	IGHV3-38-3	14q32.33	IGHV1/OR16-1	16p11.2
12	IGHV2-5	14q32.33	IGHV3/OR15-7	15q11.2	IGHV1/OR16-2	16p11.2
13	IGHV2-26	14q32.33	IGHV3/OR16-6	16p11.2	IGHV1/OR16-3	16p11.2
14	IGHV2-70	14q32.33	IGHV3/OR16-8	16p11.2	IGHV1/OR16-4	16p11.2
15	IGHV2-70D	14q32.33	IGHV3/OR16-9	16p11.2	IGHV2-10	14q32.33
16	IGHV3-7	14q32.33	IGHV3/OR16-10	16p11.2	IGHV3-6	14q32.33
17	IGHV3-9	14q32.33	IGHV3/OR16-12	16p11.2	IGHV3-19	14q32.33
18	IGHV3-11	14q32.33	IGHV3/OR16-13	16p11.2	IGHV3-22	14q32.33
19	IGHV3-13	14q32.33	IGHV4/OR15-8	15q11.2	IGHV3-29	14q32.33
20	IGHV3-15	14q32.33	IGHV7-81	14q32.33	IGHV3-30-2	14q32.33
21	IGHV3-20	14q32.33			IGHV3-30-22	14q32.33
22	IGHV3-21	14q32.33			IGHV3-30-33	14q32.33
23	IGHV3-23	14q32.33			IGHV3-30-42	14q32.33
24	IGHV3-23D	14q32.33			IGHV3-30-52	14q32.33
25	IGHV3-30	14q32.33			IGHV3-32	14q32.33
26	IGHV3-30-3	14q32.33			IGHV3-33-2	14q32.33
27	IGHV3-30-5	14q32.33			IGHV3-36	14q32.33
28	IGHV3-33	14q32.33			IGHV3-37	14q32.33
29	IGHV3-43	14q32.33			IGHV3-41	14q32.33
30	IGHV3-43D	14q32.33			IGHV3-42	14q32.33
31	IGHV3-48	14q32.33			IGHV3-42D	14q32.33
32	IGHV3-49	14q32.33			IGHV3-47	14q32.33
33	IGHV3-53	14q32.33			IGHV3-50	14q32.33
34	IGHV3-64	14q32.33			IGHV3-52	14q32.33
35	IGHV3-64D	14q32.33			IGHV3-54	14q32.33
36	IGHV3-66	14q32.33			IGHV3-57	14q32.33
37	IGHV3-72	14q32.33			IGHV3-60	14q32.33
38	IGHV3-73	14q32.33			IGHV3-62	14q32.33
39	IGHV3-74	14q32.33			IGHV3-63	14q32.33
40	IGHV3-NL1	14q32.33			IGHV3-65	14q32.33
41	IGHV4-4	14q32.33			IGHV3-69-1	14q32.33
42	IGHV4-28	14q32.33			IGHV3-71	14q32.33
43	IGHV4-30-1	14q32.33			IGHV3-75	14q32.33
44	IGHV4-30-2	14q32.33			IGHV3-76	14q32.33
45	IGHV4-30-4	14q32.33			IGHV3-79	14q32.33
46	IGHV4-31	14q32.33			IGHV3/OR16-7	16p11.2
47	IGHV4-34	14q32.33			IGHV3/OR16-11	16p11.2
48	IGHV4-38-2	14q32.33			IGHV3/OR16-14	16p11.2
49	IGHV4-39	14q32.33			IGHV3/OR16-15	16p11.2
50	IGHV4-59	14q32.33			IGHV3/OR16-16	16p11.2
51	IGHV4-61	14q32.33			IGHV4-55	14q32.33
52	IGHV5-10-1	14q32.33			IGHV4-80	14q32.33
53	IGHV5-51	14q32.33			IGHV5-78	14q32.33
54	IGHV6-1	14q32.33			IGHV7-27	14q32.33
55	IGHV7-4-1	14q32.33			IGHV7-34-1	14q32.33
56					IGHV7-40	14q32.33
57					IGHV7-40D	14q32.33
58					IGHV7-56	14q32.33
59					IGHV7-NL1	14q32.33

Human IGHD genes (IMGT database)

	Functional gene (F)*		Open reading frame (ORF)[#]		Pseudogene (P)[†]	
1	IGHD1-1	14q32.33	IGHD1-14	14q32.33	-	
2	IGHD1-7	14q32.33	IGHD1/OR15-1a	15q11.2		
3	IGHD1-20	14q32.33	IGHD1/OR15-1b	15q11.2		
4	IGHD1-26	14q32.33	IGHD2/OR15-2a	15q11.2		
5	IGHD2-2	14q32.33	IGHD2/OR15-2b	15q11.2		
6	IGHD2-8	14q32.33	IGHD3/OR15-3a	15q11.2		
7	IGHD2-15	14q32.33	IGHD3/OR15-3b	15q11.2		
8	IGHD2-21	14q32.33	IGHD4-11	14q32.33		
9	IGHD3-3	14q32.33	IGHD4-23	14q32.33		
10	IGHD3-9	14q32.33	IGHD4/OR15-4a	15q11.2		
11	IGHD3-10	14q32.33	IGHD4/OR15-4b	15q11.2		
12	IGHD3-16	14q32.33	IGHD5-24	14q32.33		
13	IGHD3-22	14q32.33	IGHD5/OR15-5a	15q11.2		
14	IGHD4-4	14q32.33	IGHD5/OR15-5b	15q11.2		
15	IGHD4-17	14q32.33				
16	IGHD5-5	14q32.33				
17	IGHD5-12	14q32.33				
18	IGHD5-18	14q32.33				
19	IGHD6-6	14q32.33				
20	IGHD6-13	14q32.33				
21	IGHD6-19	14q32.33				
22	IGHD6-25	14q32.33				
23	IGHD7-27	14q32.33				

Human IGKV genes (IMGT database)

	Functional gene (F)*		Open reading frame (ORF)[#]		Pseudogene (P)[†]	
1	IGKV1-5	2p11.2	IGKV1-37	2p11.2	IGKV1-22	2p11.2
2	IGKV1-6	2p11.2	IGKV1/OR2-0	2p11.2	IGKV1-32	2p11.2
3	IGKV1-8	2p11.2	IGKV1/OR2-108	2q12-2q14	IGKV1-35	2p11.2
4	IGKV1-9	2p11.2	IGKV1D-37	2p11.2	IGKV1/OR1-1	1 pter-1qter
5	IGKV1-12	2p11.2	IGKV1D-42	2p11.2	IGKV1/OR-2	-
6	IGKV1-13	2p11.2	IGKV2D-24	2p11.2	IGKV1/OR-3	-
7	IGKV1-16	2p11.2	IGKV3-7	2p11.2	IGKV1/OR-4	-
8	IGKV1-17	2p11.2	IGKV3/OR2-268	2p12	IGKV1-OR10-1	10q11.21
9	IGKV1-27	2p11.2	IGKV6D-41	2p11.2	IGKV1/OR15-118	15pter-15qter
10	IGKV1-33	2p11.2			IGKV1/OR2-1	2p11.2
11	IGKV1-39	2p11.2			IGKV1/OR2-11	2q11.2
12	IGKV1-NL1	2p11.2			IGKV1/OR2-118	2p11.1
13	IGKV1D-8	2p11.2			IGKV1/OR2-2	2p11.1
14	IGKV1D-12	2p11.2			IGKV1/OR2-3	2q11.2
15	IGKV1D-13	2p11.2			IGKV1/OR2-6	2q11.2
16	IGKV1D-16	2p11.2			IGKV1/OR2-9	2q11.2
17	IGKV1D-17	2p11.2			IGKV1/OR22-1	22q11
18	IGKV1D-33	2p11.2			IGKV1/OR22-5	22q11
19	IGKV1D-39	2p11.2			IGKV1/OR9-1	9q21.11
20	IGKV1D-43	2p11.2			IGKV1/OR9-2	9p12
21	IGKV2-24	2p11.2			IGKV1/ORY-1	Y
22	IGKV2-28	2p11.2			IGKV1D-22	2p11.2
23	IGKV2-29	2p11.2			IGKV1D-27	2p11.2
24	IGKV2-30	2p11.2			IGKV1D-32	2p11.2
25	IGKV2-40	2p11.2			IGKV1D-35	2p11.2
26	IGKV2D-26	2p11.2			IGKV2-4	2p11.2
27	IGKV2D-28	2p11.2			IGKV2-10	2p11.2
28	IGKV2D-29	2p11.2			IGKV2-14	2p11.2
29	IGKV2D-30	2p11.2			IGKV2-18	2p11.2
30	IGKV2D-40	2p11.2			IGKV2-19	2p11.2
31	IGKV3-11	2p11.2			IGKV2-23	2p11.2
32	IGKV3-15	2p11.2			IGKV2-26	2p11.2
33	IGKV3-20	2p11.2			IGKV2-36	2p11.2
34	IGKV3D-7	2p11.2			IGKV2-38	2p11.2
35	IGKV3D-11	2p11.2			IGKV2/OR2-1	2q11.2
36	IGKV3D-15	2p11.2			IGKV2/OR2-2	2q11.2
37	IGKV3D-20	2p11.2			IGKV2/OR2-4	2q11.2
38	IGKV4-1	2p11.2			IGKV2/OR2-7	2q11.2
39	IGKV5-2	2p11.2			IGKV2/OR2-7D	2q11.2
40	IGKV6-21	2p11.2			IGKV2/OR2-8	2q11.2
41	IGKV6D-21	2p11.2			IGKV2/OR2-10	2q11.2
42					IGKV2/OR22-3	22q11
43					IGKV2/OR22-4	22q11
44					IGKV2D-10	2p11.2
45					IGKV2D-14	2p11.2
46					IGKV2D-18	2p11.2
47					IGKV2D-19	2p11.2
48					IGKV2D-23	2p11.2
49					IGKV2D-36	2p11.2
50					IGKV2D-38	2p11.2
51					IGKV3-25	2p11.2
52					IGKV3-31	2p11.2
53					IGKV3-34	2p11.2
54					IGKV3/OR2-5	2q11.2
55					IGKV3/OR22-2	22q11
56					IGKV3D-25	2p11.2
57					IGKV3D-31	2p11.2
58					IGKV3D-34	2p11.2
59					IGKV7-3	2p11.2

***Functional gene (F)**: The coding region of the germline entity has an open reading frame without stop codon, and no described defect in the splicing sites, recombination signals and/or regulatory elements.

[#]**Open Reading Frame (ORF)**: The coding region of the germline entity has an open reading frame, but may also harbor: (i) alterations in the splicing sites, recombination signals and/or regulatory elements; (ii) and/or changes of conserved amino acids that have been suggested by authors to lead to uncorrect folding; (iii) and/or the entity is an orphon.

[†]**Pseudogene (P)**: The coding region of the germline entity has stop codon(s) and/or frameshift mutation(s). In particular, a V-GENE is considered as pseudogene if these defects occur in the L-PART1 and/or V-EXON, or if there is a mutation in the L-PART1 INIT-CODON atg.

Supplementary Table S4

Detection of IGHV genes (functional and ORF) and IGHD genes (functional and ORF) on 14q32, and IGKV genes (functional and ORF) on chromosome 2p11 with NGS primers

IGHV		PBMC				Tonsil			
IMGT Gene	Gene function	Lab A	Lab B	Lab C	Lab D	Lab A	Lab B	Lab C	Lab D
IGHV1-69	F	2.17	2.14	0.82	0.62	2.70	2.39	1.10	0.49
IGHV1-18	F	1.73	2.82	1.05	0.39	2.61	1.88	1.06	0.37
IGHV1-2	F	4.27	3.12	5.51	2.05	1.31	1.26	3.36	0.57
IGHV1-24	F	0.27	0.44	0.18	0.02	0.82	0.56	0.32	0.15
IGHV1-3	F	0.57	0.48	0.18	0.15	1.14	1.01	0.53	0.33
IGHV1-45	F	0.00	0.03	0.02	0.00	0.04	0.05	0.00	0.00
IGHV1-46	F	1.65	1.56	0.51	0.17	1.54	1.15	0.57	0.29
IGHV1-58	F	0.41	0.22	0.67	0.19	0.24	0.27	0.31	0.05
IGHV1-69-2	F	0.14	0.17	0.05	0.13	0.28	0.20	0.10	0.04
IGHV1-69D	F	2.17	2.14	0.82	0.37	2.70	2.39	1.10	0.49
IGHV1-8	F	0.79	1.23	0.82	0.42	1.08	0.73	0.45	0.19
IGHV2-26	F	0.03	1.23	2.66	0.17	0.73	0.82	1.32	0.30
IGHV2-5	F	0.59	0.62	1.63	0.20	1.15	1.19	1.33	0.20
IGHV2-70D	F	0.51	0.25	0.47	0.26	1.14	1.83	1.65	0.38
IGHV3-13	F	1.08	1.03	4.78	0.62	1.07	1.19	1.29	1.32
IGHV3-15	F	2.07	1.24	2.27	1.17	1.78	2.65	2.49	2.13
IGHV3-21	F	4.89	4.81	5.17	7.87	5.77	5.50	6.62	8.96
IGHV3-23	F	5.94	3.09	1.56	5.13	3.93	3.12	3.05	5.66
IGHV3-23D	F	5.94	3.09	1.56	5.13	3.93	3.12	3.05	5.66
IGHV3-30	F	3.89	3.73	4.40	4.24	4.19	4.11	5.20	5.86
IGHV3-30-3	F	3.89	3.73	4.40	4.24	4.19	4.11	5.20	5.86
IGHV3-30-5	F	2.85	3.70	2.37	4.09	3.44	4.07	4.87	5.06
IGHV3-33	F	2.54	0.75	2.35	2.36	0.49	0.41	0.44	0.31
IGHV3-43	F	1.33	0.35	0.98	1.72	0.34	0.36	0.50	0.64
IGHV3-43D	F	0.00	0.02	0.01	0.06	0.23	0.21	0.06	0.07
IGHV3-48	F	4.89	4.81	5.17	7.87	5.77	5.50	6.62	8.96
IGHV3-49	F	1.55	2.47	1.51	1.22	1.70	1.71	1.37	1.20
IGHV3-53	F	2.83	1.58	2.85	3.24	1.06	1.08	1.40	1.92
IGHV3-64	F	3.16	6.11	4.06	5.14	3.08	3.40	3.70	3.96
IGHV3-64D	F	0.01	0.22	0.01	0.00	0.63	0.96	0.89	0.58
IGHV3-66	F	0.03	0.45	0.85	0.66	0.92	0.75	1.05	0.56
IGHV3-7	F	4.89	4.81	5.17	7.87	5.77	5.50	6.62	8.96
IGHV3-72	F	0.00	1.04	0.51	0.54	0.52	0.55	0.46	0.59
IGHV3-73	F	0.01	0.68	0.00	0.08	0.38	0.29	0.50	0.03
IGHV3-74	F	1.58	4.93	0.60	2.25	2.65	2.76	3.07	3.75
IGHV3-9	F	6.12	3.23	2.86	6.32	3.22	3.20	3.22	4.60
IGHV3-NL1	F	2.85	3.70	2.37	4.09	3.44	4.07	4.87	5.06
IGHV4-28	F	0.00	0.50	0.46	0.19	0.04	0.06	0.02	0.03
IGHV4-30-1	F	2.67	1.05	2.21	1.63	0.82	1.10	0.70	1.17
IGHV4-30-2	F	2.22	1.99	2.08	1.32	1.13	1.18	0.78	0.95
IGHV4-30-4	F	1.49	0.71	0.44	0.87	0.91	0.94	0.56	1.02
IGHV4-31	F	2.67	1.05	2.21	1.63	0.82	1.10	0.70	1.17
IGHV4-34	F	4.08	4.16	3.52	8.39	4.28	5.38	3.36	5.69
IGHV4-38-2	F	2.42	0.24	1.24	0.84	1.02	1.20	0.68	1.12
IGHV4-39	F	5.57	5.51	5.61	6.69	3.22	2.68	1.58	2.56
IGHV4-4	F	1.51	0.34	0.98	0.92	0.91	0.87	0.51	0.58
IGHV4-59	F	3.14	3.05	1.08	1.01	2.28	1.84	1.34	1.37
IGHV5-10-1	F	0.44	0.28	1.23	0.01	1.24	0.97	1.87	0.06
IGHV5-51	F	2.33	3.67	4.82	0.51	3.10	3.59	4.43	0.35
IGHV6-1	F	0.54	0.76	0.91	0.10	0.88	0.65	1.08	0.15
IGHV7-4-1	F	0.61	0.63	0.21	0.06	0.10	0.16	0.03	0.33
IGHV2-70	F	0.58	0.14	0.35	0.01	1.14	1.83	1.65	0.38
IGHV3-20	F	0.70	1.73	0.74	0.58	0.73	0.86	0.76	1.51

IGHV4-61	F	1.15	0.42	0.08	0.24	1.07	1.24	0.87	0.93
IGHV3-11	F	0.04	0.68	0.68	1.89	0.10	0.12	0.04	0.08
IGHV1-38-4	ORF	0.17	0.18	0.45	0.00	0.01	0.02	0.22	0.04
IGHV3-16	ORF	0.00	0.00	0.00	0.00	0.00	0.00	0.00	0.00
IGHV3-35	ORF	0.00	0.00	0.00	0.00	0.04	0.07	0.04	0.04
IGHV3-38	ORF	0.00	0.19	0.00	0.00	0.01	0.01	0.00	0.00
IGHV3-38-3	ORF	0.17	0.01	0.27	0.00	0.02	0.01	0.09	0.05
IGHV7-81	ORF	0.00	0.00	0.00	0.09	0.03	0.08	0.02	0.00
IGHV3-25	ORF P	0.32	0.28	0.00	0.00	0.03	0.05	0.01	0.06

The percentages of detected genes are shown, range: 0-0.01% (white color); 0.01%-0.1% (light blue color); 0.1%-3% (middle blue color); >3% (dark blue color)

IGHD		PBMC				Tonsil			
**IMGT Gene**	**Gene function**	**Lab A**	**Lab B**	**Lab C**	**Lab D**	**Lab A**	**Lab B**	**Lab C**	**Lab D**
IGHD1-1	F	1.27	1.05	0.57	0.16	0.39	0.28	0.51	0.13
IGHD1-20	F	2.35	0.68	0.67	0.32	0.67	0.43	0.07	0.21
IGHD1-26	F	2.35	0.68	0.67	0.07	1.82	5.09	2.89	4.67
IGHD1-7	F	1.20	0.65	0.89	0.32	0.89	1.97	1.45	1.53
IGHD2-15	F	1.39	1.19	0.59	2.88	10.63	1.05	0.34	2.57
IGHD2-2	F	10.43	3.83	0.69	5.40	21.79	2.03	0.71	4.79
IGHD2-21	F	3.07	0.82	0.04	1.93	4.08	0.41	0.11	1.24
IGHD2-8	F	0.83	0.18	0.06	0.27	4.00	0.30	0.11	0.97
IGHD3-10	F	2.25	0.96	1.75	0.57	4.13	10.43	11.55	10.92
IGHD3-16	F	1.38	1.06	1.09	1.86	2.61	4.77	6.21	5.31
IGHD3-22	F	1.24	11.83	12.81	17.40	4.87	10.30	10.42	10.90
IGHD3-3	F	3.81	5.07	14.35	6.50	8.59	16.31	20.97	18.11
IGHD3-9	F	3.40	24.02	9.46	10.01	8.64	15.27	14.19	9.64
IGHD4-17	F	0.12	0.13	0.20	0.02	0.23	1.67	1.11	0.41
IGHD4-4	F	0.16	0.26	0.11	0.02	0.03	0.04	0.04	0.03
IGHD5-12	F	2.20	0.87	0.89	0.52	0.57	1.73	0.89	0.83
IGHD5-18	F	24.67	2.10	3.27	2.76	3.88	0.38	0.57	0.90
IGHD5-5	F	12.68	1.54	1.76	1.97	3.88	0.38	0.57	0.90
IGHD6-13	F	0.74	2.14	2.03	0.12	1.25	1.30	1.62	0.17
IGHD6-19	F	0.21	0.94	0.59	0.15	0.42	0.48	0.39	0.04
IGHD6-25	F	0.32	3.94	6.75	0.47	1.03	1.99	1.21	0.19
IGHD6-6	F	0.94	2.54	0.96	0.20	1.45	1.35	1.77	0.25
IGHD1-14	ORF	1.02	0.82	0.31	0.35	0.40	0.39	0.26	0.30
IGHD4-11	ORF	2.22	0.56	0.51	0.18	0.33	0.09	0.09	0.04
IGHD4-23	ORF	2.22	0.56	0.51	0.18	0.53	0.55	1.23	0.28
IGHD5-24	ORF	31.15	2.69	7.41	10.94	6.19	2.15	2.47	5.37

The percentages of detected genes are shown, range: 0-0.01% (white color); 0.01%-0.1% (light blue color); 0.1%-3% (middle blue color); >3% (dark blue color)

**IGKV**		**PBMC**				**Tonsil**			
**IMGT Gene**	**Gene function**	**Lab A**	**Lab B**	**Lab C**	**Lab D**	**Lab A**	**Lab B**	**Lab C**	**Lab D**
IGKV1-12	F	0.49	0.81	0.16	1.63	0.71	0.56	0.59	1.35
IGKV1-17	F	3.30	2.67	1.80	4.74	1.69	1.66	1.14	2.68
IGKV1-27	F	3.71	3.71	2.33	5.56	2.24	2.63	1.88	4.16
IGKV1-33	F	4.80	4.44	3.09	3.53	6.04	5.09	3.99	5.84
IGKV1-5	F	4.72	5.43	1.87	7.46	4.86	4.13	2.71	7.19
IGKV1-6	F	0.99	0.73	0.63	0.71	0.57	0.33	0.30	0.70
IGKV1-8	F	3.68	2.36	1.26	3.05	1.59	1.52	1.04	2.62
IGKV1-9	F	0.72	1.48	0.69	1.62	0.92	0.77	0.56	1.55
IGKV1D-12	F	0.36	0.36	0.12	0.95	0.95	0.82	0.62	1.53
IGKV1D-13	F	0.74	1.05	0.42	1.06	1.82	1.67	1.19	2.95
IGKV1D-16	F	2.46	2.83	1.82	2.85	3.20	2.90	1.95	3.63
IGKV1D-17	F	3.30	2.67	1.80	4.74	1.69	1.66	1.14	2.68
IGKV1D-33	F	4.80	4.44	3.09	3.53	6.04	5.09	3.99	5.84
IGKV1D-39	F	6.21	6.53	6.10	18.76	9.51	9.10	8.20	20.33
IGKV1D-43	F	1.20	0.92	0.68	0.49	1.19	1.34	0.83	0.84
IGKV1D-8	F	1.57	1.35	1.21	3.87	0.96	0.92	1.01	1.81
IGKV1-NL1	F	0.13	0.03	0.21	1.39	0.26	0.14	0.18	1.36
IGKV2-24	F	1.87	1.64	1.05	1.09	1.74	1.48	1.24	1.55
IGKV2-28	F	6.19	1.89	1.14	2.09	6.65	2.15	1.60	1.76
IGKV2-40	F	0.38	0.44	0.77	0.69	1.04	0.88	1.15	1.30
IGKV2D-26	F	4.61	3.13	6.71	2.58	5.37	5.02	7.78	3.18
IGKV2D-28	F	6.19	1.89	1.14	2.09	6.65	2.15	1.60	1.76
IGKV2D-29	F	1.84	1.26	3.56	0.98	0.68	0.60	1.07	0.39
IGKV2D-30	F	5.98	6.66	5.49	4.00	7.44	6.81	6.15	5.28
IGKV2D-40	F	0.32	0.71	1.17	1.16	1.04	0.88	1.15	1.30
IGKV3-11	F	3.53	4.49	4.32	2.05	3.23	4.22	3.89	2.60
IGKV3-15	F	2.71	3.68	2.84	1.95	2.70	3.67	3.14	1.85
IGKV3-20	F	6.82	7.09	7.47	3.02	6.60	9.19	9.28	3.65
IGKV3D-11	F	0.82	0.51	0.49	0.57	0.63	0.57	0.58	0.48
IGKV3D-7	F	1.66	1.59	0.91	1.02	0.81	0.99	0.78	0.47
IGKV4-1	F	5.82	6.99	15.35	0.86	5.96	8.96	15.65	0.77
IGKV5-2	F	4.49	3.17	4.64	4.51	2.80	2.67	2.36	4.77
IGKV6-21	F	0.13	0.60	0.87	0.77	0.22	0.39	0.71	1.09
IGKV6D-21	F	0.14	0.41	1.26	0.36	0.22	0.39	0.71	1.09
IGKV1-16	F [F]	2.46	2.83	1.82	2.85	3.20	2.90	1.95	3.63
IGKV2-30	F [F]	3.08	3.49	3.45	1.64	7.44	6.81	6.15	5.28
IGKV3D-20	F ORF	6.82	7.09	7.47	3.02	6.60	9.19	9.28	3.65
IGKV1-13	F P	0.74	1.05	0.42	1.06	1.82	1.67	1.19	2.95
IGKV1-39	F P	6.21	6.53	6.10	18.76	9.51	9.10	8.20	20.33
IGKV2-29	F P	1.07	1.87	3.68	1.40	3.21	3.62	4.48	1.92
IGKV3D-15	F P	2.71	3.68	2.84	1.95	2.70	3.67	3.14	1.85
IGKV1-37	ORF	3.43	2.81	4.40	5.70	3.90	4.19	5.18	6.43
IGKV1D-37	ORF	3.43	2.81	4.40	5.70	3.90	4.19	5.18	6.43
IGKV1D-42	ORF	0.00	0.00	0.00	0.00	0.00	0.00	0.00	0.00
IGKV2D-24	ORF	0.36	0.92	0.68	0.74	0.20	0.19	0.15	0.17
IGKV6D-41	ORF	0.15	0.11	0.68	0.00	0.08	0.04	0.07	0.18
IGKV3-7	ORF [ORF]	1.66	1.59	0.91	1.02	0.42	0.45	0.45	0.24
IGKV7-3	P#	1.07	2.51	1.56	0.82	0.61	0.60	0.45	0.31
Intron		6.71	9.81	5.15	0.59	6.54	7.11	4.77	0.96

The percentages of detected genes are shown, range: 0-0.01% (white color); 0.01%-0.1% (light blue color); 0.1%-3% (middle blue color); >3% (dark blue color)

# IGKV7-3 is annotated as pseudogene, but frequently found rearranged in our dataset and therefore included in the list

Supplementary Table S5

Lab A (Nijmegen; NIJ)

Sample	Total reads	Mapped reads (%)	Mapped reads for IGHV-IGHD-IGHJ	Mapped reads for IGHD-IGHJ	Mapped reads for IGKV-IGKJ + IGKV/Intron-KDE
Tonsil 1-Frozen	82136	66966 (82%)	36848 (55%)	4768 (7%)	25350 (38%)
Tonsil 2-FFPE	108145	94968 (88%)	34087 (36%)	12518 (13%)	48363 (51%)
Tonsil 3-Frozen	110624	92729 (84%)	50613 (55%)	14965 (16%)	27151 (29%)
Tonsil 4-FFPE	127574	109425 (86%)	37981 (35%)	17107 (16%)	54337 (49%)

Lab B (Rotterdam; ROT)

Sample	Total reads	Mapped reads (%)	Mapped reads for IGHV-IGHD-IGHJ	Mapped reads for IGHD-IGHJ	Mapped reads for IGKV-IGKJ + IGKV/Intron-KDE
Tonsil 1-Frozen	179056	152101 (85%)	61286 (40%)	26596 (18%)	64219 (42%)
Tonsil 2-FFPE	40476	35221 (87%)	18403 (52%)	6824 (20%)	9994 (28%)
Tonsil 3-Frozen	56722	48912 (86%)	29157 (60%)	11336 (23%)	8419 (17%)
Tonsil 4-FFPE	122314	104851 (86%)	49149 (47%)	17930 (17%)	37772 (36%)

Lab C (Berlin; BER)

Sample	Total reads	Mapped reads (%)	Mapped reads for IGHV-IGHD-IGHJ	Mapped reads for IGHD-IGHJ	Mapped reads for IGKV-IGKJ + IGKV/Intron-KDE
Tonsil 1-Frozen	204038	153701 (75%)	57045 (37%)	13475 (9%)	83181 (54%)
Tonsil 2-FFPE	214758	182747 (85%)	45426 (25%)	15733 (9%)	121588(66%)
Tonsil 3-Frozen	191263	152688 (80%)	60016 (39%)	16800 (11%)	75872 (50%)
Tonsil 4-FFPE	224374	186974 (83%)	38220 (21%)	17607 (9%)	131147(70%)

Lab D (Tübingen; TUB)

Sample	Total reads	Mapped reads (%)	Mapped reads for IGHV-IGHD-IGHJ	Mapped reads for IGHD-IGHJ	Mapped reads for IGKV-IGKJ + IGKV/Intron-KDE
Tonsil 1-Frozen	76487	50846 (66%)	2662 (5%)	11561 (23%)	36623 (72%)
Tonsil 2-FFPE	113767	73314 (64%)	1487 (2%)	22580 (31%)	49427 (67%)
Tonsil 3-Frozen	105545	72953 (69%)	3880 (5%)	19034 (26%)	50039 (69%)
Tonsil 4-FFPE	55447	34588 (62%)	561 (2%)	12669 (36%)	21358 (62%)

Supplementary Table S6

Representation of IGHV, IGHD and IGKV genes in tonsil DNA as determined in four different centres

	Lab A		Lab B		Lab C		Lab D	
**IGHV percentages in Tonsil tissue_1**	**Frozen**	**FFPE**	**Frozen**	**FFPE**	**Frozen**	**FFPE**	**Frozen**	**FFPE**
IGHV1-2	1.63	1.95	4.64	3.09	1.58	3.35	0.75	1.68
IGHV1-3	0.69	0.77	0.40	0.58	0.83	0.75	0.19	0.00
IGHV1-8	0.72	1.01	0.47	0.27	1.27	1.09	0.30	0.27
IGHV1-18	1.68	1.59	0.85	0.45	2.74	2.23	0.38	0.20
IGHV1-46	1.04	1.20	0.49	0.41	1.50	0.33	0.30	0.13
IGHV1-69	1.48	0.78	0.73	0.59	1.60	2.07	0.23	0.20
IGHV1-69-2	0.11	0.13	0.08	0.06	0.30	0.04	0.00	0.00
IGHV2-5	1.37	1.31	1.15	1.66	1.02	0.60	0.26	0.20
IGHV2-26	0.61	0.56	1.48	1.20	0.43	1.03	0.34	0.00
IGHV2-70	1.83	1.16	1.72	2.51	0.87	0.94	0.30	0.67
IGHV3-7	3.10	3.56	3.68	3.98	2.36	3.04	4.06	3.77
IGHV3-9	2.81	1.92	2.95	2.66	3.26	2.57	3.91	4.44
IGHV3-11	2.93	2.85	3.80	2.53	2.35	2.85	2.89	4.57
IGHV3-13	0.88	1.36	0.96	0.77	1.08	0.94	1.16	2.82
IGHV3-15	2.74	2.12	2.77	3.85	2.04	2.14	2.07	4.10
IGHV3-20	0.54	1.00	0.43	0.27	0.45	0.60	0.68	0.07
IGHV3-23	2.86	3.16	1.96	3.11	3.49	5.06	5.15	4.37
IGHV3-30	1.37	3.42	2.65	3.05	2.01	2.62	2.67	3.70
IGHV3-49	1.76	1.71	1.61	1.73	2.04	1.07	1.09	1.41
IGHV3-53	0.80	1.50	1.09	1.22	0.74	0.83	1.39	0.81
IGHV3-64D	1.34	0.99	1.25	2.52	0.68	1.78	0.75	1.01
IGHV3-66	1.21	1.66	1.70	2.56	1.52	1.35	0.86	0.54
IGHV3-72	0.54	0.48	0.41	0.85	0.60	0.40	0.75	0.20
IGHV3-73	0.29	0.90	0.75	0.59	0.47	0.56	0.04	0.07
IGHV3-74	2.85	2.61	3.17	3.11	2.91	2.67	3.79	5.45
IGHV4-34	6.55	6.49	3.74	4.57	4.59	4.16	5.79	3.30
IGHV4-38-2	1.05	1.58	0.40	1.26	0.79	0.61	1.24	1.48
IGHV4-39	5.16	5.81	2.93	4.80	6.12	4.69	5.00	4.30
IGHV4-59	2.75	1.43	2.29	1.52	3.75	1.97	1.62	1.21
IGHV4-61	1.13	0.92	0.77	0.34	0.90	0.10	0.86	0.40
IGHV5-51	3.37	3.02	4.03	3.83	2.69	1.86	0.41	0.20
IGHV6-1	0.55	1.13	1.27	1.16	1.01	1.16	0.04	0.27

The percentages of detected genes are shown, range: 0-0.01% (white color); 0.01%-0.1% (light blue color); 0.1%-3% (middle blue color); >3% (dark blue color)

	Lab A		Lab B		Lab C		Lab D	
**IGHD percentages in Tonsil tissue_1**	**Frozen**	**FFPE**	**Frozen**	**FFPE**	**Frozen**	**FFPE**	**Frozen**	**FFPE**
IGHD1-1	0.55	0.62	0.99	1.07	0.20	0.55	0.23	0.48
IGHD1-7	1.49	2.60	1.70	2.20	0.72	0.72	1.44	0.84
IGHD1-20	0.84	1.45	0.12	1.48	0.76	0.43	0.39	0.39
IGHD1-26	5.85	14.26	3.26	13.45	1.90	4.90	5.34	6.22
IGHD1/OR15-1a	2.30	2.81	5.01	3.88	2.15	3.60	2.18	3.76
IGHD2-2	2.66	0.64	0.98	0.16	22.71	25.11	4.75	3.67
IGHD2-15	0.99	0.43	0.37	0.15	8.85	11.49	2.17	2.24
IGHD3-3	16.23	14.47	23.23	16.87	8.41	9.21	18.52	26.60
IGHD3-9	13.70	11.78	12.77	10.48	7.16	7.93	8.06	7.55
IGHD3-10	10.93	7.95	10.45	6.95	3.26	6.94	8.71	11.43
IGHD3-16	4.09	3.86	6.35	6.51	1.83	3.66	5.00	5.41
IGHD3-22	11.26	13.02	11.16	15.42	5.03	7.25	12.50	10.88
IGHD3/OR15-3a	0.34	1.34	0.95	1.14	0.27	1.22	0.35	1.20
IGHD3/OR15-3b	0.34	1.34	0.95	1.14	0.27	1.22	0.35	1.20
IGHD4-17	2.03	0.75	0.76	0.26	0.24	0.01	0.38	0.19
IGHD4-23	1.36	0.89	2.68	3.09	0.45	0.41	0.90	0.74
IGHD5-5	2.68	2.83	2.27	1.03	0.54	0.67	4.18	2.64
IGHD5-12	2.14	2.27	1.06	0.69	0.30	0.20	1.65	0.80
IGHD5-24	2.62	3.12	2.44	5.45	9.31	1.25	7.25	5.84
IGHD5/OR15-5b	2.79	2.32	2.08	1.80	0.82	0.87	3.55	2.34
IGHD6-6	1.38	0.79	1.42	1.36	0.94	0.93	0.27	0.30
IGHD6-19	0.36	0.56	0.38	0.41	0.29	0.32	0.03	0.01
IGHD6-25	2.12	1.95	0.64	1.42	0.79	0.86	0.22	0.10

The percentages of detected genes are shown, range: 0-0.01% (white color); 0.01%-0.1% (light blue color); 0.1%-3% (middle blue color); >3% (dark blue color)

	Lab A		Lab B		Lab C		Lab D	
**IGKV percentages in Tonsil tissue_1**	**Frozen**	**FFPE**	**Frozen**	**FFPE**	**Frozen**	**FFPE**	**Frozen**	**FFPE**
IGKV1-5	4.62	3.60	3.00	2.09	5.49	4.99	7.54	6.41
IGKV1-8	1.53	1.96	1.02	0.83	1.62	1.54	2.75	2.73
IGKV1-9	0.49	0.88	0.36	0.27	0.71	0.58	0.98	0.64
IGKV1-27	2.40	2.81	1.67	2.48	2.22	2.89	4.29	4.95
IGKV1D-13=IGKV1-13	2.30	2.51	1.43	1.24	2.52	2.51	4.24	3.99
IGKV1D-16=IGKV1-16	3.15	2.82	1.90	1.13	3.55	3.62	4.33	2.94
IGKV1D-17=IGKV1-17	1.76	1.85	1.05	0.87	1.65	1.21	2.43	2.34
IGKV1D-33=IGKV1-33	5.28	5.89	3.99	2.99	6.36	5.94	5.83	5.37
IGKV1D-37=IGKV1-37	4.37	3.19	5.16	5.12	3.80	3.99	6.96	7.96
IGKV1D-39=IGKV1-39	9.45	8.31	7.54	6.80	9.43	12.17	19.64	23.07
IGKV1D-43	1.51	1.20	0.83	0.64	1.22	1.10	0.66	0.88
IGKV2-24	1.53	2.01	1.35	1.61	1.86	1.53	1.49	1.50
IGKV2D-29	3.83	2.90	4.15	5.99	3.20	3.14	2.05	1.30
IGKV2D-26	5.86	6.46	8.20	10.36	6.34	5.77	3.43	1.92
IGKV2D-28=IGKV2-28	2.37	0.97	2.11	0.66	6.95	4.44	1.42	1.65
IGKV2D-29	1.84	1.77	2.93	2.94	1.54	0.96	0.80	0.61
IGKV2D-30=IGKV2-30	6.22	6.47	6.64	6.76	7.61	5.92	4.23	4.94
IGKV3-11	4.38	4.81	4.41	4.58	3.34	4.04	2.80	2.55
IGKV3D-7	0.89	1.29	0.89	1.11	0.83	0.98	0.49	0.60
IGKV3D-15=IGKV3-15	4.08	4.61	3.97	3.59	3.00	3.46	2.02	1.53
IGKV3D-20=IGKV3-20	7.48	9.13	8.98	7.87	5.43	6.54	3.09	3.12
IGKV4-1	8.80	9.85	13.75	19.46	4.72	9.18	0.72	0.84
IGKV5-2	2.30	1.79	2.26	2.77	2.21	2.29	3.96	4.08
Intron	5.85	3.36	4.20	1.43	5.49	2.79	0.72	0.70

The percentages of detected genes are shown, range: 0-0.01% (white color); 0.01%-0.1% (light blue color); 0.1%-3% (middle blue color); >3% (dark blue color)

	Lab A		Lab B		Lab C		Lab D	
**IGHV percentages in Tonsil tissue_2**	**Frozen**	**FFPE**	**Frozen**	**FFPE**	**Frozen**	**FFPE**	**Frozen**	**FFPE**
IGHV1-2	0.88	0.42	2.09	5.26	1.04	1.58	0.39	0.00
IGHV1-3	1.33	1.13	0.67	0.78	1.45	0.84	0.46	0.18
IGHV1-8	0.74	0.55	0.44	0.21	0.88	0.33	0.08	1.96
IGHV1-18	2.07	1.21	1.27	0.54	2.47	2.66	0.36	0.53
IGHV1-46	1.27	1.81	0.65	0.49	1.57	1.42	0.28	0.18
IGHV1-69	2.21	1.53	1.25	0.76	2.29	2.96	0.41	0.89
IGHV1-69-2	0.28	0.54	0.12	0.00	0.27	0.70	0.08	0.00
IGHV2-5	1.00	0.43	1.51	1.79	1.27	0.21	0.13	0.00
IGHV2-26	1.04	0.00	1.16	0.97	1.03	1.47	0.26	0.00
IGHV2-70	1.82	1.81	1.57	2.32	1.40	1.39	0.46	0.18
IGHV3-7	2.91	1.32	3.48	2.82	3.19	5.12	3.56	2.50
IGHV3-9	3.59	5.02	3.48	2.00	3.17	1.88	5.28	3.39
IGHV3-11	2.95	2.25	3.91	5.87	3.15	3.43	3.27	6.06
IGHV3-13	1.50	0.35	1.62	1.95	1.05	0.09	1.47	0.89
IGHV3-15	2.56	1.93	2.21	4.42	1.52	1.54	2.19	1.96
IGHV3-20	1.18	0.66	1.09	0.04	1.01	1.08	2.35	3.21
IGHV3-23	3.34	4.34	3.32	5.23	3.31	2.96	5.62	8.02
IGHV3-30	2.69	5.27	3.35	5.17	2.55	5.74	3.12	1.78
IGHV3-49	0.20	0.01	0.24	0.01	0.32	0.52	0.13	0.00
IGHV3-53	1.36	2.76	1.72	0.17	1.39	1.19	2.45	0.89
IGHV3-64D	0.59	0.36	0.54	0.02	0.57	0.01	0.41	1.07
IGHV3-72	0.56	0.39	0.50	0.03	0.43	0.00	0.44	0.36
IGHV3-73	0.28	0.60	0.26	0.00	0.29	0.25	0.03	0.00
IGHV3-74	2.68	5.43	2.97	1.27	2.38	3.57	3.71	1.96
IGHV4-34	4.22	3.63	2.97	2.07	3.97	1.87	5.59	2.14
IGHV4-38-2	0.71	0.49	0.58	0.17	0.75	0.53	1.31	1.07
IGHV4-59	2.49	2.40	1.66	0.48	2.67	1.82	1.65	4.10
IGHV4-61	1.39	2.59	1.07	1.79	1.37	1.41	1.37	1.43
IGHV5-51	3.81	3.13	4.83	7.00	3.52	2.66	0.28	0.53
IGHV6-1	0.75	1.46	0.90	0.44	0.76	0.54	0.26	0.00

The percentages of detected genes are shown, range: 0-0.01% (white color); 0.01%-0.1% (light blue color); 0.1%-3% (middle blue color); >3% (dark blue color)

	Lab A		Lab B		Lab C		Lab D	
**IGHD percentages in Tonsil tissue_2**	**Frozen**	**FFPE**	**Frozen**	**FFPE**	**Frozen**	**FFPE**	**Frozen**	**FFPE**
IGHD1-1	1.34	0.37	1.21	0.71	0.57	0.36	0.67	0.07
IGHD1-7	2.45	2.78	1.21	2.20	1.05	0.29	1.62	1.66
IGHD1-20	0.75	0.92	0.53	0.83	0.32	0.59	0.49	1.25
IGHD1-26	4.33	7.15	2.51	4.90	1.75	1.55	4.00	2.46
IGHD1/OR15-1a	0.96	1.32	0.50	0.89	0.46	0.18	0.36	0.59
IGHD2-2	1.40	0.75	0.44	0.28	20.86	21.01	4.83	4.47
IGHD2-15	1.11	0.60	0.30	0.31	12.40	13.96	2.97	2.95
IGHD3-3	16.39	15.63	18.71	26.37	8.77	10.84	17.71	17.69
IGHD3-9	16.85	16.41	15.61	11.82	10.13	7.76	11.21	9.84
IGHD3-10	9.92	9.35	12.66	10.82	4.99	6.40	13.13	17.07
IGHD3-16	5.45	6.08	6.08	4.58	3.39	4.20	5.62	5.38
IGHD3-22	9.34	12.65	9.69	11.99	4.71	7.66	9.29	14.53
IGHD/OR15-3a	3.15	2.43	4.66	2.50	3.10	3.49	4.31	2.57
IGHD/OR15-3b	3.15	2.43	4.66	2.50	3.10	3.49	4.31	2.57
IGHD4-17	1.31	0.98	1.46	0.37	0.21	0.03	0.45	0.24
IGHD4-23	0.97	0.59	2.33	1.35	0.40	0.27	0.54	0.27
IGHD5-5	2.97	2.49	2.79	1.28	1.36	0.97	3.59	4.17
IGHD5-12	3.31	3.54	1.63	1.79	0.52	0.59	1.54	0.85
IGHD5-24	1.68	1.44	2.50	3.29	3.06	0.90	3.49	1.89
IGHD5/OR15-5b	3.91	3.78	3.72	3.50	2.03	0.93	4.78	4.03
IGHD6-6	1.32	0.59	2.13	1.76	1.97	1.18	0.23	0.13
IGHD6-19	0.61	0.94	0.39	0.46	0.56	1.79	0.04	0.02
IGHD6-25	1.86	1.27	1.77	1.30	1.27	1.36	0.16	0.06

The percentages of detected genes are shown, range: 0-0.01% (white color); 0.01%-0.1% (light blue color); 0.1%-3% (middle blue color); >3% (dark blue color)

	Lab A		Lab B		Lab C		Lab D	
**IGKV percentages in Tonsil tissue_2**	**Frozen**	**FFPE**	**Frozen**	**FFPE**	**Frozen**	**FFPE**	**Frozen**	**FFPE**
IGKV1-5	3.64	4.64	2.42	2.42	4.23	5.64	6.84	6.11
IGKV1-8	1.50	2.13	1.07	0.96	1.56	1.55	2.49	2.10
IGKV1-9	1.05	0.54	0.75	0.76	1.13	1.17	2.12	2.53
IGKV1-27	2.85	3.71	2.08	2.91	2.26	3.87	4.03	4.75
IGKV1D-13=IGKV1-13	1.05	1.06	0.94	0.73	1.12	1.27	1.65	0.94
IGKV1D-16=IGKV1-16	2.65	1.97	2.00	1.18	2.86	2.20	2.93	4.10
IGKV1D-17=IGKV1-17	1.56	2.01	1.22	0.82	1.73	2.56	2.94	4.03
IGKV1D-33=IGKV1-33	4.90	4.66	3.99	3.95	5.73	6.20	5.84	3.82
IGKV1D-37=IGKV1-37	4.00	3.19	5.20	8.32	3.99	6.58	5.90	4.05
IGKV1D-39=IGKV1-39	8.76	9.28	8.86	9.64	9.59	7.97	21.02	24.50
IGKV1D-43	1.18	0.98	0.82	0.91	1.16	1.20	1.03	1.12
IGKV2-24	1.42	2.67	1.14	0.73	1.62	1.64	1.61	1.65
IGKV2-29	3.40	3.54	4.81	5.28	3.22	2.77	1.79	2.17
IGKV2D-26	4.19	4.65	7.36	8.19	4.41	5.75	2.93	3.03
IGKV2D-29	1.17	1.35	1.92	0.65	1.24	1.14	0.75	0.48
IGKV2D-30=IGKV2-30	7.40	5.09	5.67	6.83	7.28	3.97	6.33	6.51
IGKV3-11	4.06	6.11	3.36	4.40	3.11	3.19	2.41	1.48
IGKV3D-7	1.09	1.62	0.67	0.39	0.79	0.73	0.45	0.70
IGKV3D-15=IGKV3-15	3.27	4.99	2.32	4.22	2.41	2.97	1.68	1.41
IGKV3D-20=IGKV3-20	10.90	8.54	9.57	9.58	7.76	8.97	4.21	3.54
IGKV4-1	9.12	10.88	17.54	14.61	7.19	10.45	0.82	2.02
IGKV5-2	3.05	4.48	2.46	4.15	3.39	3.14	5.58	7.17
Intron	8.37	4.56	5.35	2.16	7.60	4.55	1.20	0.72

The percentages of detected genes are shown, range: 0-0.01% (white color); 0.01%-0.1% (light blue color); 0.1%-3% (middle blue color); >3% (dark blue color)

Supplementary Table S7

Results NGS-based clonality assessment B cell lymphoma samples - detection of the most abundant clonotype

B cell lymphoma sample	Tumor type at diagnosis*	Lab A or C				Lab B or D			
		IGHV-IGHD-IGHJ	IGHD-IGHJ	IGKV-IGKJ	IGKV/Intron-KDE	IGHV-IGHD-IGHJ	IGHD-IGHJ	IGKV-IGKJ	IGKV/Intron-KDE
Blym1	DLBCL abdomen	IGHV3-23(D)/IGHJ4	IGHD2-2/IGHJ6	IGKV1(D)-39/IGKJ4	IGKV2(D)-30/KDE Intron/KDE	IGHV3-23(D)/IGHJ4	IGHD2-2/IGHJ6	IGKV1(D)-39/IGKJ4	IGKV2(D)-30/KDE Intron/KDE
Blym2	EBV+ PTLD	IGHV4-30-4=V4-61/IGHJ6# IGHV4-34=V4-59/IGHJ6#	IGHD2-2/IGHJ5	IGK4-1/IGKJ4 IGKV3(D)-7=V3/OR2-68/IGKJ5	Intron-KDE (2x)	IGHV4-30-4=V4-61/IGHJ6# IGHV4-34=V4-59/IGHJ6#	IGHD2-2/IGHJ5	IGK4-1/IGKJ4 IGKV3(D)-7=V3/OR2-268/IGKJ5	Intron-KDE (2x)
Blym3	Cutaneous DLBCL	Multiple clonotypes	IGHD2-2/IGHJ6	IGKV2-29/IGKJ3 IGKV2-29/IGKJ2	Intron/KDE (2x)	Multiple clonotypes	IGHD2-2/IGHJ6	IGKV2-29/IGKJ3 IGKV2-29/IGKJ2	Intron/KDE (2x)
Blym4	Gastrointestinal MZL	IGHV3-48/IGHJ3	IGHD3-22/IGHJ6	IGKV1(D)-33/IGKVJ3 IGKV3(D)-20/IGKJ3	Intron/KDE	IGHV3-48/IGHJ3	IGHD3-22/IGHJ6	IGKV1(D)-33/IGKVJ3 IGKV3(D)-20/IGKJ3	Intron/KDE
Blym5	Anaplastic plasmacytoma; extraosseous	IGHV3-7=V3-11=V3-21=V3-48=V3-69-1=V3-74/IGHJ3	IGHD2-2/IGHJ6	IGKV3(D)-15/IGKJ4	IGKV1(D)-16/KDE Intron-KDE	IGH3-7=V3-11=V3-21=V3-48=V3-69-1=V3-74/IGHJ3	IGHD2-2/IGHJ6	IGKV3(D)-15/IGKJ4	IGKV1(D)-16/KDE Intron-KDE
Blym6	EBV+ PTLD	IGHV5-78/IGHJ4	IGHD5-12/IGHJ6# IGHD5/OR15-5b/IGHJ6#	IGKV1(D)-39/IGKJV2	Intron/KDE	IGHV5-78/IGHJ4 IGHV3-NL1=V3-30-3=V3-30-5=V3-30/IGHJ4	IGHD5-12/IGHJ6# IGHD5/OR15-5b/IGHJ6#	IGKV1(D)-39/IGKJV2	Intron/KDE
Diag1	Uterine cervix DLBCL	IGHV4-34/ IGHJ4	IGHD5-24/IGHJ2	IGKV5-2/IGKJ2 IGKV1D-8/IGKJ4	Intron/KDE (2 x)	IGHV4-34/ IGHJ4	IGHD5-24/IGHJ2	IGKV5-2/IGKJ2 IGKV1D-8/IGKJ4	Intron/KDE (2 x)
Relap1	Vermiform appendix DLBCL	IGHV4-34/ IGHJ4	IGHD5-24/IGHJ2	IGKV5-2/IGKJ2 IGKV1D-8/IGKJ4	Intron/KDE (2 x)	IGHV4-34/ IGHJ4	IGHD5-24/IGHJ2	IGKV5-2/IGKJ2 IGKV1D-8/IGKJ4	Intron/KDE (2 x)
Diag2	Testicular DLBCL	Multiple clones	IGHD2-21/IGHJ3	IGKV2-29/IGKJ3 IGKV3-11/IGKJ5	Intron-KDE (2 x)	Weak IGHV3-23(D)/IGHJ4	IGHD2-21/IGHJ3	IGKV2-29/IGKJ3 IGKV3-11/IGKJ5	Intron-KDE (2 x)
Relap2	CLL/SLL bone marrow	IGHV3-23(D)/IGHJ4	IGHD6-6/IGHJ4	IGKV4-1/IGKJ5	IGKV1-12/KDE Intron-KDE	IGHV3-23(D)/IGHJ4	IGHD6-6/IGHJ4	IGKV4-1/IGKJ5	IGKV1-12/KDE Intron-KDE
Diag3	Nodal cervical MZL	Polyclonal	Polyclonal	Polyclonal VJ	IGKV1-5/KDE	Polyclonal	Polyclonal	Polyclonal VJ	IGKV1-5/KDE
Relap3	Nodal cervical MZL	Polyclonal	Weak IGHD6-6/IGHJ5	Weak IGKV3(D)/IGKJ1	IGKV1-5/KDE	Polyclonal	Polyclonal	Polyclonal VJ	IGKV1-5/KDE
Diag4	Glandula parotis DLBCL	IGHV3-23/IGHJ4	IGHD2-15/IGHJ4	Polyclonal VJ	IGKV2(D)-30/KDE Intron/KDE	Not evaluable (very few reads)	IGHD2-15/IGHJ4	Polyclonal VJ	IGKV2(D)-30/KDE Intron/KDE
Relap4	Extranodal lung MZL	IGHV3-15/IGHJ4 +PCB	IGHD2-15/IGHJ6	IGK V3(D)-20IGKJ2=J3 +PCB	Multiple clonotypes	IGHV3-48/IGHJ4 +PCB	IGHD2-15/IGHJ6 +PCB	Multiple clonotypes	IGKV1D-37/KDE

* Abbreviations used: DLBCL, Diffuse large B cell lymphoma; EBV, Epstein-Barr virus; MZL, marginal zone lymphoma; PTLD, post-transplant lymphoproliferative disorder; CLL, chronic lymphocytic leukemia; SLL, small lymphocytic lymphoma; PCB, polyclonal background.
# Related clonotypes belonging most likely to one rearrangement; single nucleotide variations probably due to somatic hypermutation or sequencing artifacts.

<u>Supplementary Table S8</u>

Summary performance next-generation sequencing (NGS)-based clonality assessment
Comparison between the methodologies (EuroClonality/BIOMED-2 assay with GeneScan (GS) analysis and NGS), and inter-lab variation related to the multicentre study for NGS-based clonality assessment, in identifying the dominant clonotype within the B cell lymphoma specimen.

Samples	**IGHV-IGHD-IGHJ**		**IGHD-IGHJ**		**IGKV-IGKJ**		**IGKV/Intron-KDE**	
	NGS-GS	Lab 1-Lab 2	NGS-GS	Lab 1-Lab 2	NGS-GS	Lab 1-Lab 2	NGS-GS	Lab 1-Lab 2
Blym1								
Blym2								
Blym3	#1							
Blym4								
Blym5								
Blym6								
Diag1								
Relap1								
Diag2								
Relap2								
Diag3								
Relap3								
Diag4								
Relap4					#2		#2	

Concordance between GeneScan/ NGS and Lab 1-Lab 2
Improved performance of NGS versus GeneScan: detection R by NGS not detected by GS
Discordance between NGS and GeneScan, inferior performance NGS vs GS
Discordance between Lab 1-Lab 2, mostly due to variable efficiency multiplex PCR

#1: Mismatches probably due to somatic hypermutation on template of NGS IGHV primer
#2: NGS detected a broader range of multiple clonotypes

SUPPLEMENTARY FIGURES AND LEGENDS

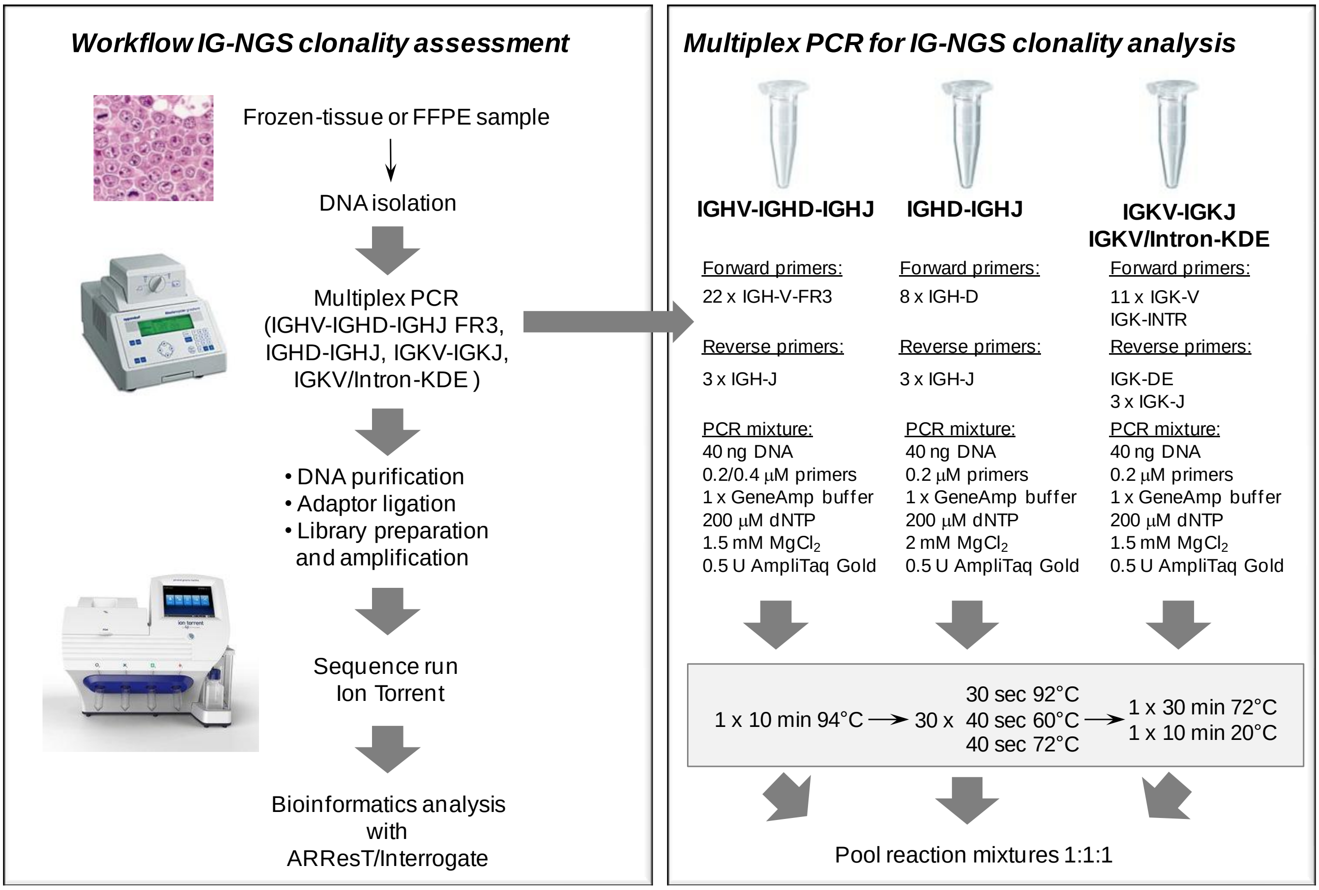

Supplementary Figure S1. Schematic overview of the workflow for detecting immunoglobulin rearrangements for clonality assessment by next-generation sequencing. The left panel indicates the different steps of the workflow, starting with the DNA isolation of tissue specimens, either frozen-tissue or formalin-fixed paraffin-embedded (FFPE) sample, followed by multiplex PCR for the detection of IGHV-IGHD-IGHJ, IGHD-IGHJ, IGKV-IGKJ and IGKV/Intron-KDE gene rearrangements. The right panel shows the details of the multiplex PCR, including the amount of forward and reverse primers, the composition of the PCR reaction mixture and the PCR amplification conditions. After the individual PCR reactions are mixed at similar ratios, the PCR amplicons are purified, adaptors are ligated and a pooled library is prepared and amplified. Subsequently, the samples are sequenced on an Ion Torrent machine followed by bioinformatics analyses with ARResT/Interrogate.

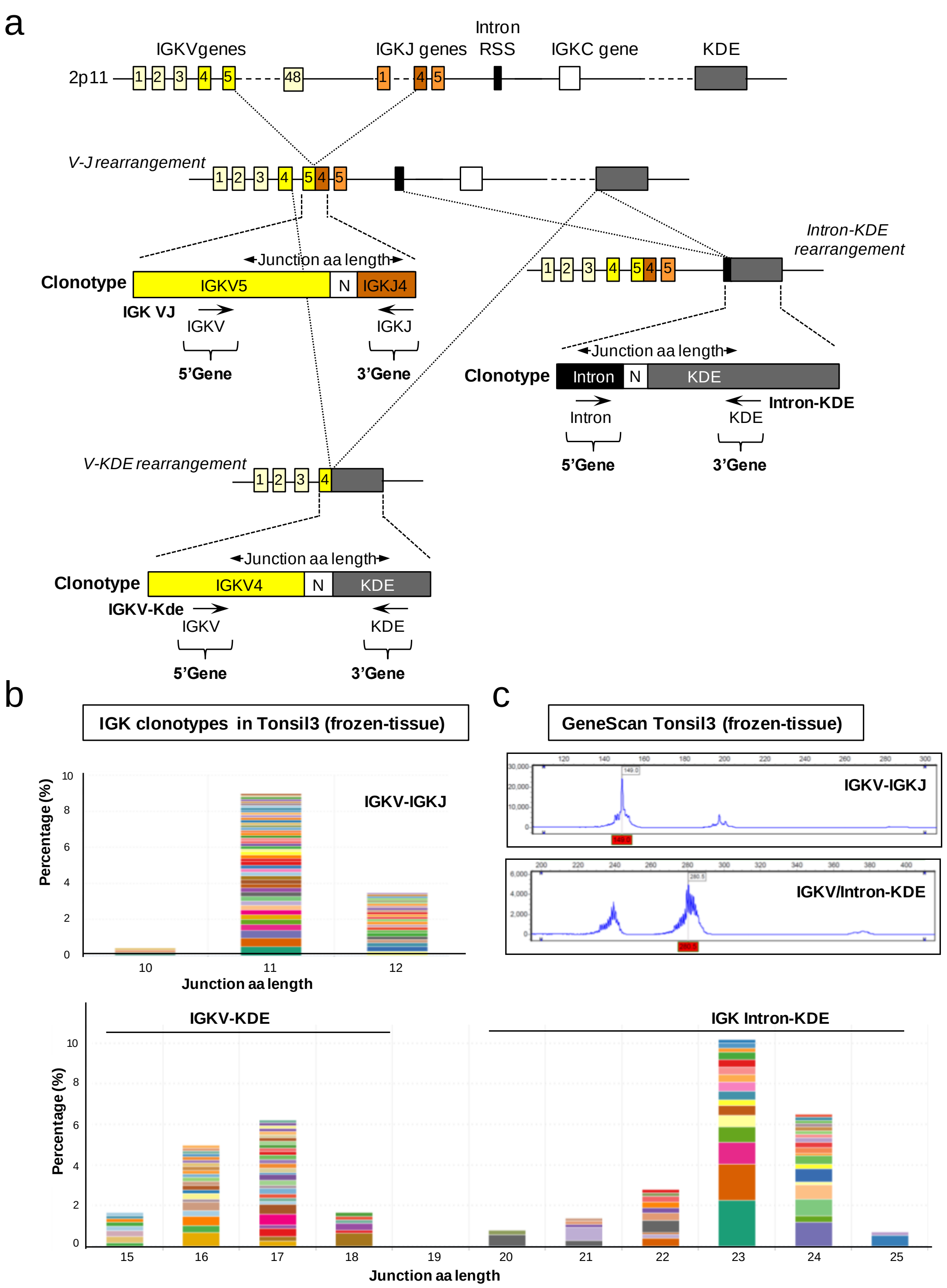

Supplementary Figure S2. Identification of distinct clonotypes by next-generation sequencing (NGS)-based detection of immunoglobulin kappa light chain gene rearrangements. (**a**) Schematic representation of the immunoglobulin kappa light chain (IGK) locus on chromosome 2p11, indicating the presence of 48 IGKV genes, 5 IGKJ genes and one IGKC gene. Within the intron between IGKJ and IGKC genes, there is an active heptamer recombination signal sequence (RSS) and a downstream κ-deleting (Kde) element. Following RAG-mediated IGKV-IGKJ, Intron-KDE and/or IGKV-KDE locus gene rearrangement, each B cell can generate up to four distinct IGK clonotypes. For the detection of IGKV-IGKJ gene rearrangements,

forward IGKV and reverse IGKJ primers will generate amplicons. For the detection of Intron-KDE rearrangements, forward Intron and reverse KDE primers yield products, while for IGKV-KDE locus rearrangements, forward IGKV and reverse primer KDE will generate amplicons. Successful amplification will generate fragments that cover junctional regions with a given amino acid (aa) length. (**b**) Detection of distinct clonotypes in DNA isolated from Tonsil3 (frozen-tissue) of a healthy donor, indicating a polyclonal pattern of IGKV-IGKJ and IGKV/Intron-KDE rearrangements that encode variable aa length at the junction between IGKV and IGKJ, and IGKV or Intron and KDE (see accompanying manuscript by Knecht et al, submitted). Each coloured bar indicates a distinct clonotype, and the 100 most abundant clonotypes are shown as analyzed by ARResT/Interrogate. Most of the IGKV-KDE clonotypes are depicted at the left side, while the majority of IGKV/Intron-KDE clonotypes are present at the right side. (**c**) GeneScan profile of Tonsil3 for IGKV-IGKJ and IGKV/Intron-KDE using EuroClonality/BIOMED-2 PCR.

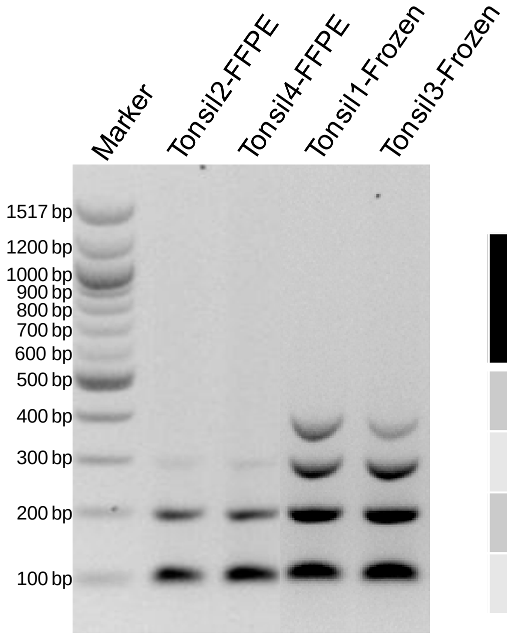

Sample	DNA quality (PCR)	KAPA qPCR Q129/ Q41	Q305/ Q41
Tonsil1-Frozen	400 bp	0.83	0.82
Tonsil2-FFPE	300w bp	0.27	0.02
Tonsil3-Frozen	400 bp	0.69	0.54
Tonsil4-FFPE	300w bp	0.31	0.01

Supplementary Figure S3. DNA quality control of tonsil specimens isolated from frozen-tissue and formalin-fixed paraffin-embedded (FFPE) material. DNA quality PCR analysis was performed on genomic DNA; 300w, 300 weak intensity band. Next to size ladder PCR, the DNA quality was determined by qPCR according to the KAPA Human genomic DNA quantification and QC Kit. The ratio between the 129 bp and 41 bp fragments and 305 bp and 41 bp fragments are indicated.

a

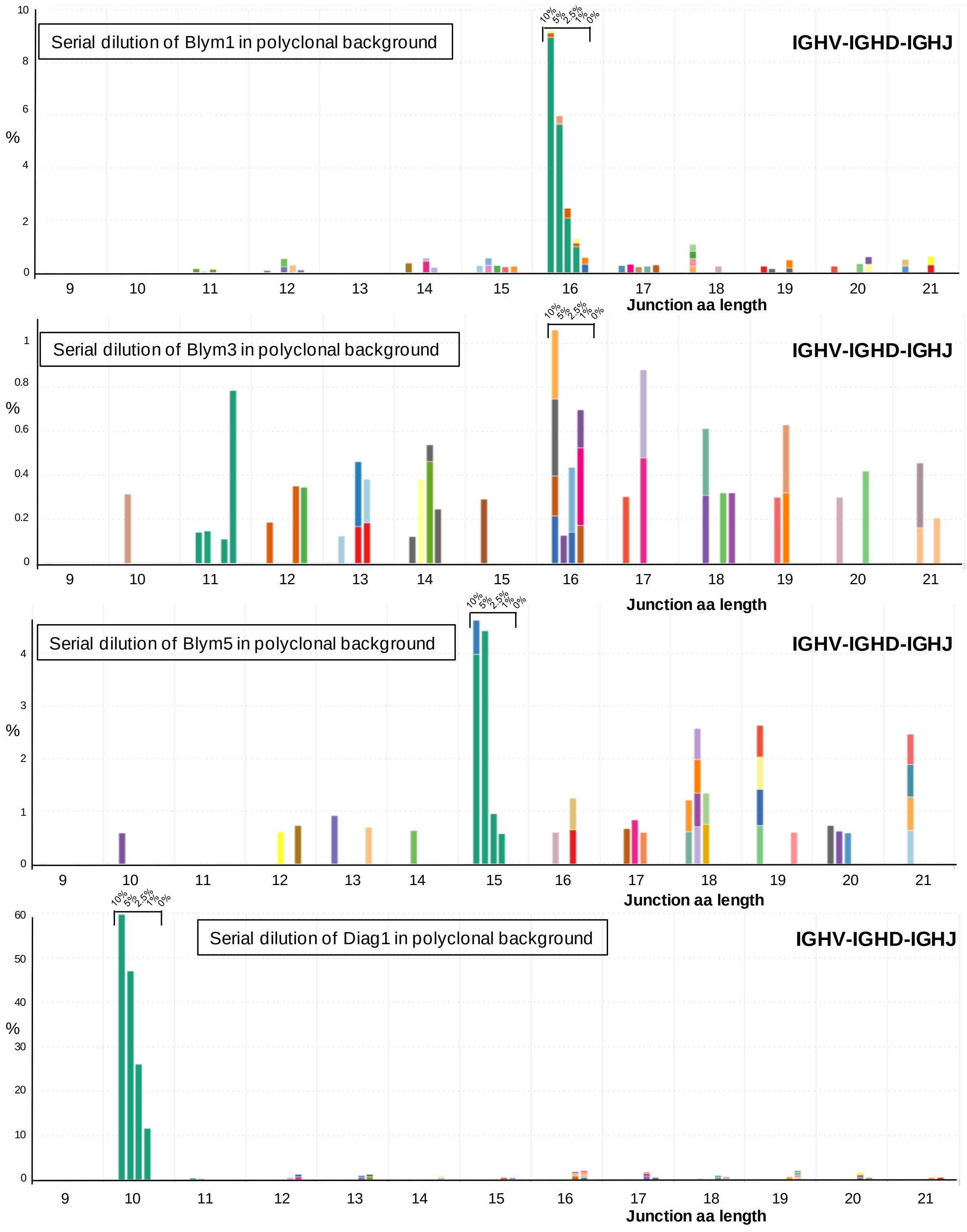
Serial dilution of Blym1 in polyclonal background
IGHV-IGHD-IGHJ
10% 5% 2.5% 1% 0%
%
Junction aa length
Serial dilution of Blym3 in polyclonal background
IGHV-IGHD-IGHJ
10% 5% 2.5% 1% 0%
%
Junction aa length
Serial dilution of Blym5 in polyclonal background
IGHV-IGHD-IGHJ
10% 5% 2.5% 1% 0%
%
Junction aa length
Serial dilution of Diag1 in polyclonal background
IGHV-IGHD-IGHJ
10% 5% 2.5% 1% 0%
%
Junction aa length

b

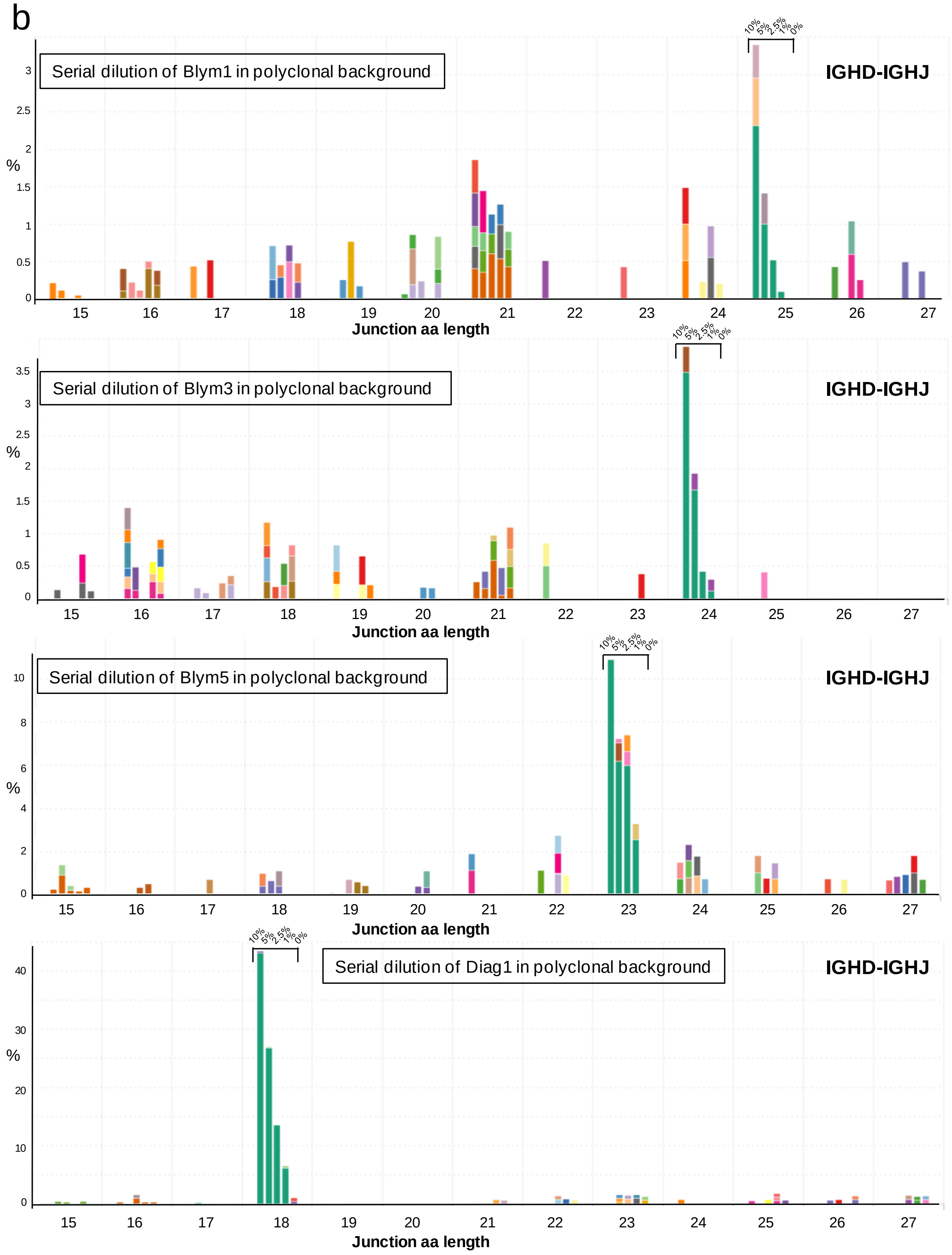

Serial dilution of Blym1 in polyclonal background
IGHD-IGHJ
10% 5% 2.5% 1% 0%
%
Junction aa length
Serial dilution of Blym3 in polyclonal background
IGHD-IGHJ
10% 5% 2.5% 1% 0%
%
Junction aa length
Serial dilution of Blym5 in polyclonal background
IGHD-IGHJ
10% 5% 2.5% 1% 0%
%
Junction aa length
Serial dilution of Diag1 in polyclonal background
IGHD-IGHJ
10% 5% 2.5% 1% 0%
%
Junction aa length

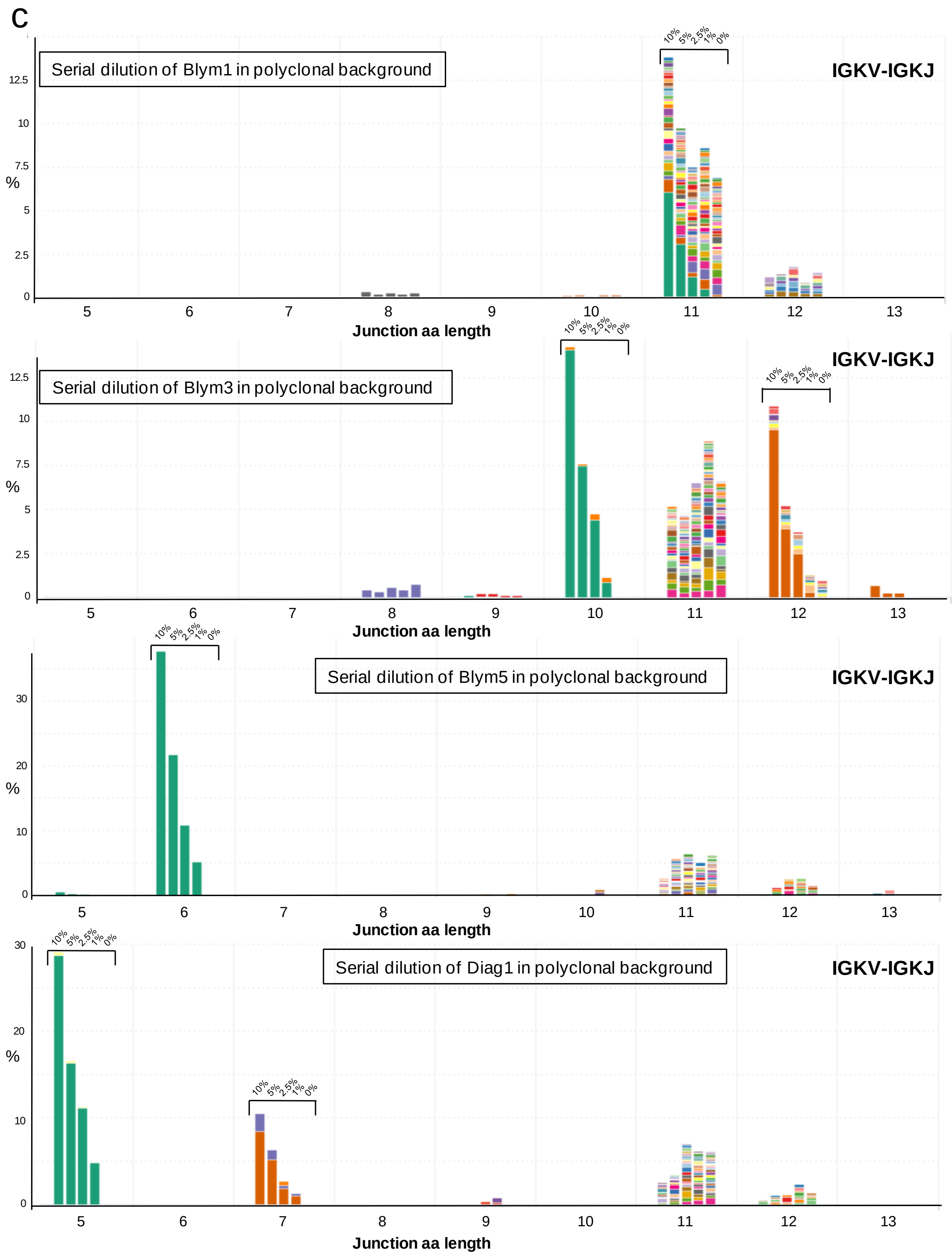

Supplementary Figure S4. Sensitivity of next-generation sequencing to detect clonal immunoglobulin gene rearrangements in a polyclonal background. (a-c) DNA of four B cell lymphoma samples (Blym1, Blym3, Blym5 and Diag1) with high tumor load (80%-90%) were serially diluted with DNA extracted from formalin-fixed paraffin (FFPE)-embedded tonsil specimen, at a final concentration of 10%, 5%, 2.5%, 1% and 0% tumor DNA. The specific clonotypes detected for IGHV-IGHD-IGHJ (a), IGHD-IGHJ (b), and IGKV-IGKJ (c) are indicated. In all cases, except for IGHV-IGHD-IGHJ gene rearrangements in sample Blym3, specific clonotypes could be detected at 10% tumor DNA, while at lower percentages this was evident for some of the samples.

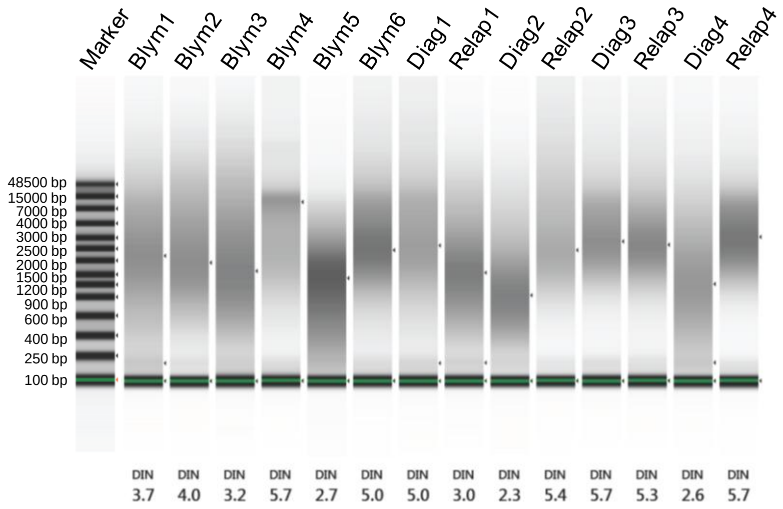

Sample	Q129/Q41	Q305/Q41
Blym1	0.32	0.08
Blym2	0.34	0.04
Blym3	0.29	0.01
Blym4	0.82	0.45
Blym5	0.50	0.12
Blym6	0.51	0.19
Diag1	0.50	0.11
Relap1	0.34	0.03
Diag2	0.33	0.04
Relap2	0.65	0.12
Diag3	0.62	0.23
Relap3	0.57	0.27
Diag4	0.05	0.00
Relap4	0.56	0.26

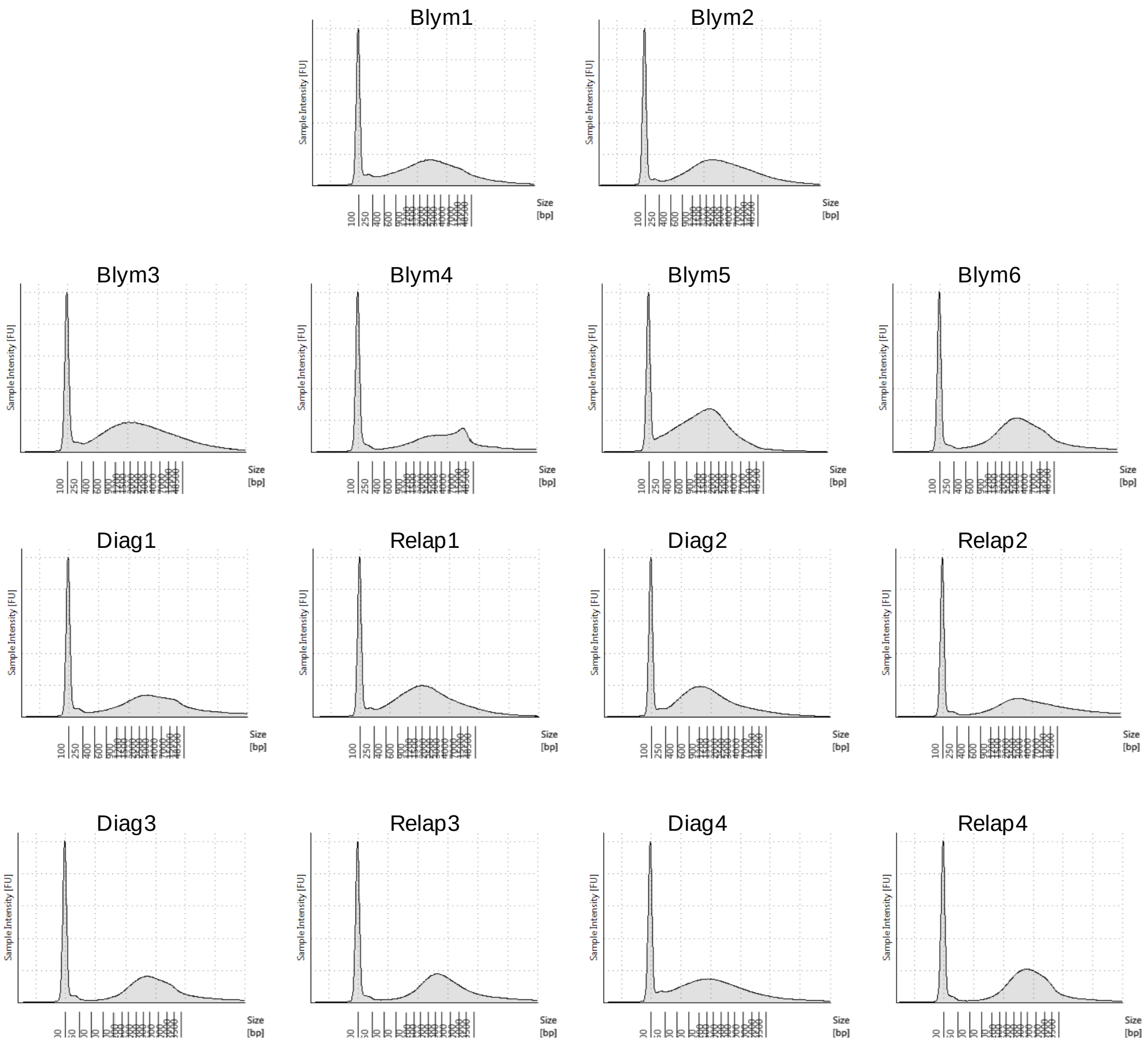

Supplementary Figure S5. DNA quality control of B cell lymphoma specimens isolated from formalin-fixed paraffin-embedded material. Genomic DNA ScreenTape with Agilent 2200 TapeStation System, and KAPA qPCR were used to assess the DNA quality. DIN: DNA integrity number.

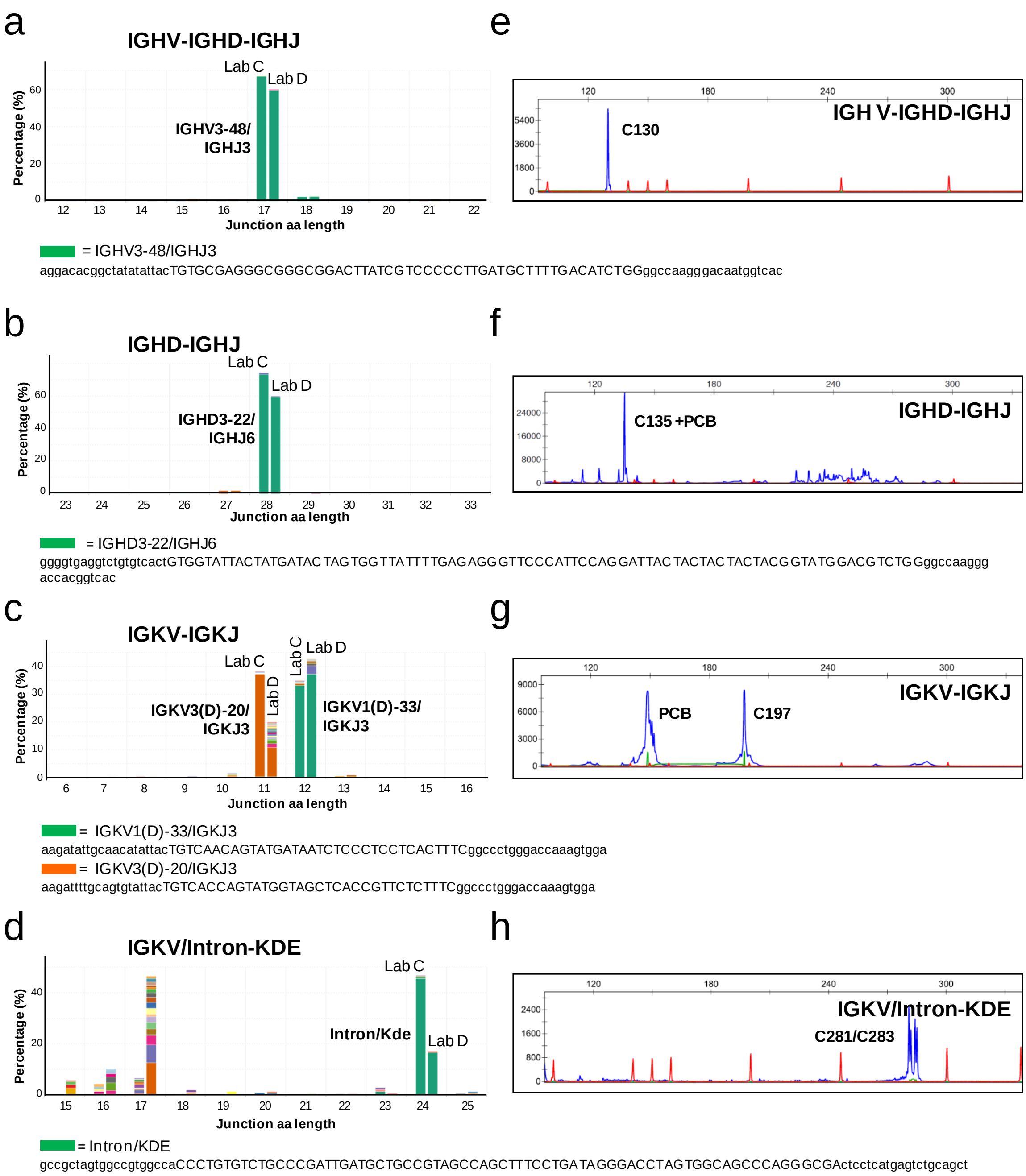

Supplementary Figure S6. Detection of additional IGK locus gene rearrangements by next-generation sequencing (NGS)-based clonality analysis. B cell lymphoma specimen Blym4 was analyzed for IGH (IGHV-IGHD-IGHJ and IGHD-IGHJ) and IGK (IGKV-IGKJ and IGKV/Intron-KDE) gene rearrangements by NGS (a-d) in two independent centres (Lab C and Lab D) and compared to EuroClonality/BIOMED-2 assay (e-f). The sequence of the abundant clonotypes is shown, with the nucleotide junction sequence in capitals (see accompanying manuscript by Knecht et al., this issue) and flanking sequences in lower case, which have been trimmed to 20 nucleotides length. PCB: polyclonal background. In sample Blym4, NGS allowed detection of bi-allelic IGK VJ rearrangements, which were not detected by EuroClonality/BIOMED-2 primers.

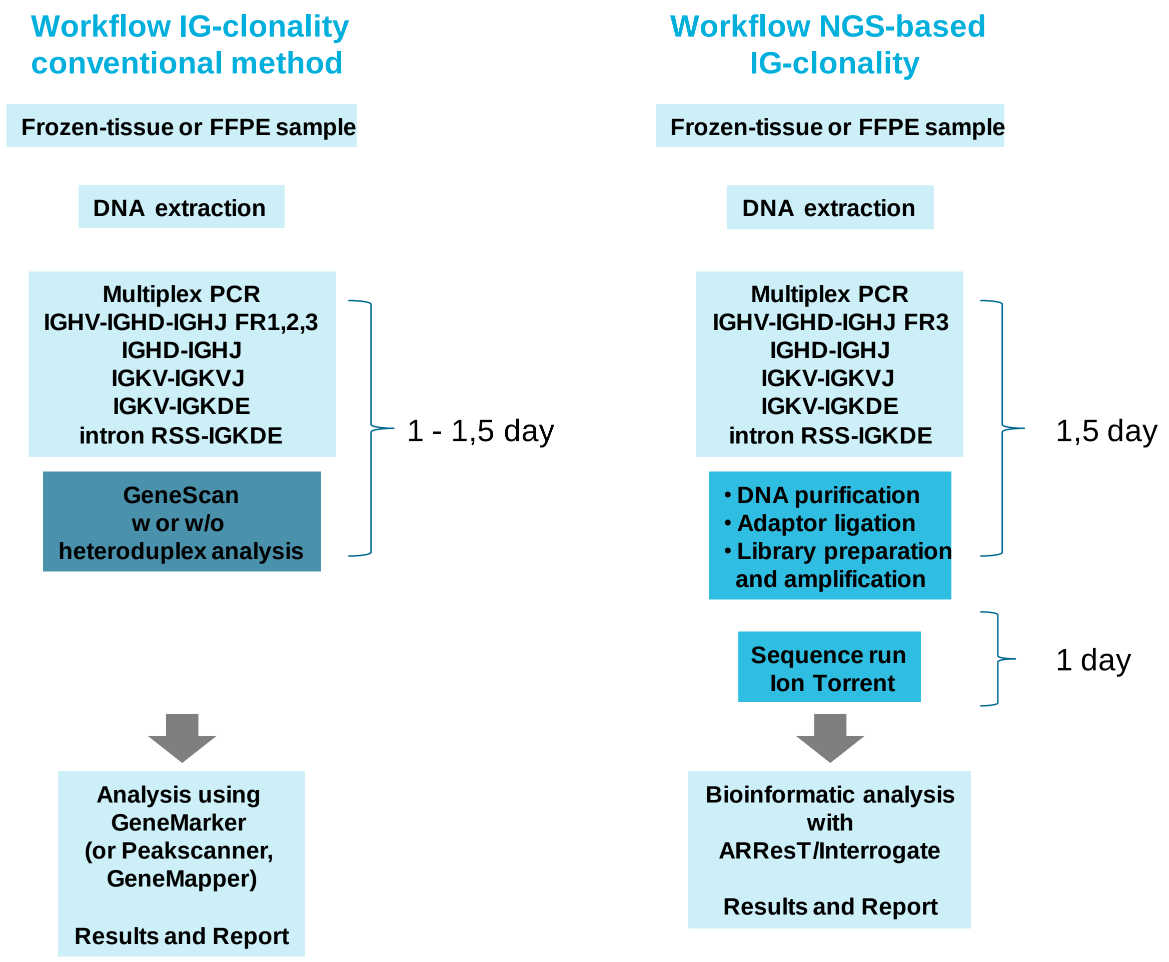

Supplementary Figure S7. Schematic representation of the turnaround time comparing conventional GeneScan analysis with next-generation sequencing (NGS)-based clonality. The workflow for the conventional method of clonality analysis involving the EuroClonality/BIOMED-2 approach with GeneScan is indicated on the left side and has a turnaround time of 1-1,5 days excluding the analysis time. NGS-based clonality, as indicated on the right side, has an estimated turnaround time of 2,5 days for the wet-lab part and sequencing time, followed by the bioinformatics analysis.
